# Differential Gene Expression and Epiregulation of Alpha Zein Gene Copies in Maize Haplotypes

**DOI:** 10.1371/journal.pgen.1002131

**Published:** 2011-06-23

**Authors:** Mihai Miclaus, Jian-Hong Xu, Joachim Messing

**Affiliations:** Waksman Institute of Microbiology, Rutgers, The State University of New Jersey, Piscataway, New Jersey, United States of America; The University of North Carolina at Chapel Hill, United States of America

## Abstract

Multigenic traits are very common in plants and cause diversity. Nutritional quality is such a trait, and one of its factors is the composition and relative expression of storage protein genes. In maize, they represent a medium-size gene family distributed over several chromosomes and unlinked locations. Two inbreds, B73 and BSSS53, both from the Iowa Stiff Stock Synthetic collection, have been selected to analyze allelic and non-allelic variability in these regions that span between 80–500 kb of chromosomal DNA. Genes were copied to unlinked sites before and after allotetraploidization of maize, but before transposition enlarged intergenic regions in a haplotype-specific manner. Once genes are copied, expression of donor genes is reduced relative to new copies. Epigenetic regulation seems to contribute to silencing older copies, because some of them can be reactivated when endosperm is maintained as cultured cells, indicating that copy number variation might contribute to a reserve of gene copies. Bisulfite sequencing of the promoter region also shows different methylation patterns among gene clusters as well as differences between tissues, suggesting a possible position effect on regulatory mechanisms as a result of inserting copies at unlinked locations. The observations offer a potential paradigm for how different gene families evolve and the impact this has on their expression and regulation of their members.

## Author Summary

We present here how the structure and function of a multigene family has shaped the architecture of the maize genome in a haplotype-specific manner, before and after allotetraploidization. The alpha zein gene family, the main component of storage protein genes, provides us with a model of how multicopy gene families evolve and are regulated in the plant kingdom. Indeed, gene copying might be the mechanism that helps plants adapt to variable environmental conditions. In this context, the alpha zein genes have evolved from a common ancestral copy, located on the short arm of chromosome 1, to become a 41-member gene family in the reference maize genome, B73. Different haplotypes can vary, though, as we show here, both in gene copy number and in their sequence context, the latter one being the result of the tremendous transposable element activity that the maize genome has undergone after its allotetraploidization. That had impact not only on the expression patterns of the gene family members, with newest copies contributing the most of the mRNA pool, but also on the mechanisms employed in their regulation, such as methylation of promoter sequences, which seems to be locus-specific.

## Introduction

Sequencing entire genomes of several plant species has shown that a prominent feature is the extensive duplications of genes [1], [2]. Because the duplication of genes is frequently associated with a change in gene regulation [3], it has been suggested that copying genes could represent a response to the environmental challenge that plants have to meet because of their immobility [4]. Therefore, it has been of great interest to determine the timing and mechanisms of gene duplications and the role of each copy in gene expression. Variation in gene copy number has also been observed between closely related species because of syntenic alignments of chromosomal regions. If a gene were copied before a progenitor of two species splits, one would expect that both copies would be present in progeny genomes. For instance, in the comparison of maize, sorghum, and rice, the *fie* gene homologs were duplicated in tandem before their progenitor split. After maize arose by a whole-genome duplication event, the sorghum and rice lineages retained both tandem copies, while the duplicated regions of the maize genome lost one of the two gene copies [5]. Indeed, a genome-wide analysis of tagged genes linked to a physical map indicated that polyploidization of maize led to massive losses of one of the duplicated gene copies [6].

Therefore, alignments between orthologous chromosomal segments of duplicated regions in maize with those of sorghum and rice have been used to examine how a single gene family has expanded before and after the polyploidization of maize [5]. In this case, the expansion and shrinkage of gene copies are tied to an important quantitative trait. Such a trait in cereal crops is the nutritional quality of their grain. The grain is the source of essential amino acids for the diet of animals and humans. Because cereal grain contains very little free amino acids, the bulk is derived from its protein content. Therefore, the relative proportion of each protein and its amino acid composition in the mature seeds of cereal crops dictates its nutritional quality [7]. Indeed, seeds accumulate proteins during maturation that have no known enzymatic function but specifically store amino acids, which are hydrolyzed during germination. These proteins are therefore called storage proteins. In cereals like rice, sorghum, and maize, they are referred to as prolamines because of their high content in proline and glutamine. They fall into four groups based on amino acid sequence homology, the α-, β-, γ-, and δ-prolamines. While in rice γ-prolamines have been extensively amplified and placed in unlinked locations [8] in sorghum and maize α-prolamines have undergone a similar expansion [9]. Because of these differences between rice and sorghum, they can act as an excellent reference to maize in respect to differential gene amplification. Interestingly, the first copying event that occurred in the progenitor of sorghum and maize resulted into a new locus, containing prolamines of two sizes: 19-kDa and 22-kDa. Additional copying occurred in each genome, but gene copies were also lost, either entirely deleted or damaged through premature stop codons. It is interesting that damaged gene copies have been quite stable over a long period of time and there is evidence that in some cases transcripts of genes with stop codons accumulate at low levels [10], [11]. The low level could be explained by turnover of aborted translation of mRNA [12]. Nevertheless, transcription per se could be the reason that the life of genes is extended despite the fact that no full-length proteins are produced. Furthermore, alleles have been found that differ only by a premature stop codon, indicating that gene conversion might counteract gene silencing [13]. Whereas mechanisms of generating paralogous gene copies are poorly understood, syntenic alignments have shown that genes can insert at close and unlinked distances. Therefore, it is likely that copying involves also an extrachromosomal copy that includes some of the flanking sequences as well, which contain common target sites for transcriptional activators [14]. In other cases, where genes are tandemly duplicated, unequal crossing over between flanking direct repeats (DR1, DR2, DR3) might have been an alternative route early in evolution [15].

To examine the role of gene copies within a multigene family, we took advantage of the haplotype variability of the α-zein gene family between two different inbred lines of maize. This family has been subdivided based on sequence homology and chromosomal location in *z1A*, *z1B*, *z1C*, and *z1D* [16]. The differential abundance is mostly based on tandem gene amplification, except that *z1A* and *z1C* gene copies are present in two locations on chromosome *4S*. Interestingly, there are also allelic differences of individual gene copies between different inbred lines. Indeed, amino acid sequence heterogeneity has been used to map individual genes by IEF-gel analysis of segregating hybrids [17]. These observations could also be explained by the presence or absence of gene copies.

There are a total of six different loci: one on chromosome 1, four on chromosome 4 (two of them physically linked), and one on chromosome 7. At each locus, copies can be spread apart by several 100 kb, requiring the cloning of overlapping chromosomal fragments. Here, we took advantage of a BAC library made from inbred BSSS53 [15] as well as a supplementary BSSS53 BAC library and screened for clones comprising the allelic chromosomal regions of B73. The BSSS53 clones were also sequenced and their content analyzed. Annotated sequences of both inbreds were aligned via their genes. Because these regions refer to a unique set of allelic differences that can be inherited as a linked unit, we consider them haplotypes of these loci. The prominent feature of these haplotypes is that they can differ in the content of sequences rather than simply single nucleotide polymorphism (SNPs). Consequently, haplotypes have diverged in intergenic spacing and gene content, mostly in recent times of less than 2 mya, but long before domestication. There is also an interesting chronological order of events, where gene insertions are followed by retrotransposition into intergenic regions and even sometimes into genes. Variability has also an impact on the accumulation of transcripts, illustrating that quantitative traits could be directly linked to non-allelic gene copies within the same species. Interestingly, when endosperm is cultured, transcription of some gene copies can be induced, indicating that their expression was epigenetically regulated.

## Results

### BAC sequencing of the α-zein genes in B73 and BSSS53 inbreds

**Figure 1:**
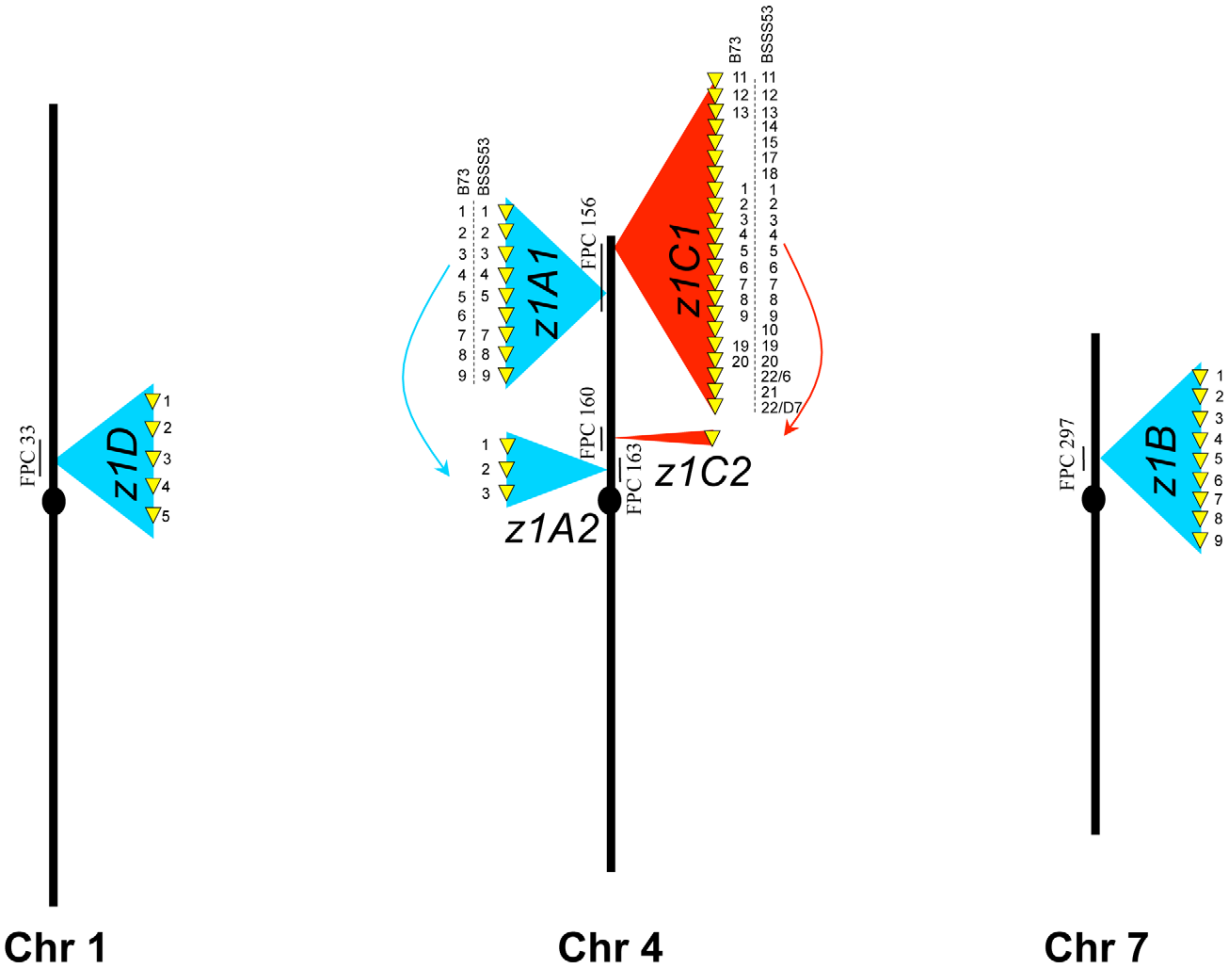
Genomic distribution of the α-zeins in the two inbred lines. Zein gene copies at each locus in the genome are presented as yellow arrows on a blue (19-kDa clusters) or red (22-kDa clusters) background. When copy number differs between the two inbreds the zeins are numbered accordingly. Each locus is anchored on the FPC map and the contig is indicated.

Taking advantage of the available FPC map [18] we positioned the *z1D* locus to FPC33 on chromosome *1S*, the *z1A1* and *z1C1* locus to FPC156 on chromosome *4S*, the *z1C2* locus to FPC160 on chromosome *4S*, the *z1A2* locus to FPC163 on chromosome *4S*, the *z1B1* and *z1B2* loci on FPC297 on chromosome *7S* (Figure 1). These placements are consistent with previous mapping experiments [11]. However, *z1B1* and *z1B2* were closer than expected from the genetic map. While hybridization experiments have failed to link clone c0492M16 representing *z1B1* and clone c0531H07 representing *z1B2* [19], both clones could be connected based on the FPC map with a single overlapping clone. From FPC297, clone b397H03, was chosen and sequenced (accession GQ214221.1) because at the time the study was underway the reference genome sequence had not been available. Furthermore, although the B73 maize genome-sequencing project provided an excellent tiling path of overlapping BAC clones, individual clones lacked contiguous sequences. Here, one contiguous sequence was formed from the three overlapping clones comprising the entire *z1B* locus. It turned out that the bridging clone added one additional member of the *z1B* gene cluster, which was not present on the flanking BAC clones. As a result, we now have a complete set of α-zein copies in B73.

**Figure 2:**
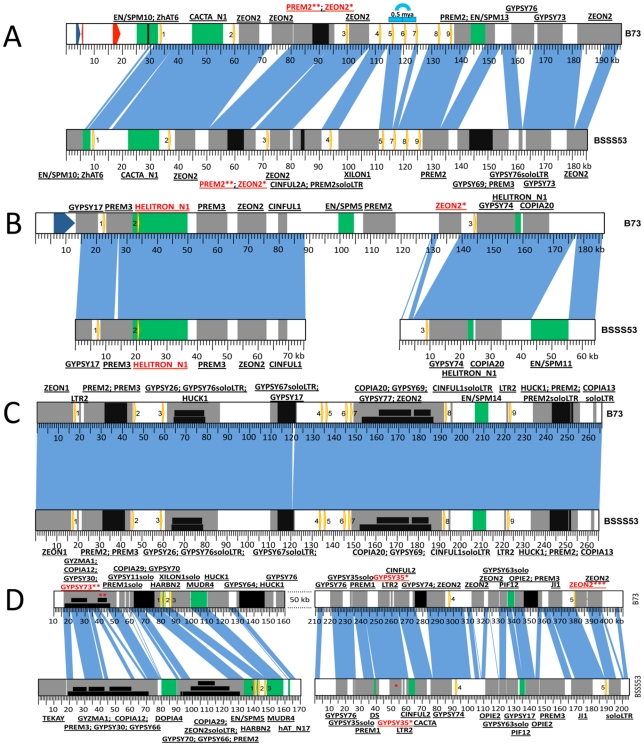
Haplotype variability at the 19-kDa loci. Zein genes are numbered from left to right, as yellow arrows. Sequence homology between the two inbreds is represented by vertical blue lines. DNA transposons are represented by green boxes while REs are in grey. Insertions in either DNA transposons or REs are depicted as black boxes. Nesting of REs is illustrated in different layers of black boxes. The first name in the label represents the original TE; the ones that follow are subsequent insertions in the order in which they occurred. (A) *Haplotype variability extends to gene copy number at the z1A1 locus.* Positioned on chromosome *4S* this locus harbors 9 zein genes in B73 and only 8 in BSSS53. The blue arch illustrates the 5 kb tandem duplication that generated the extra zein copy in B73. The two red arrows represent predicted gene models while the blue one is a putative gene present in B73. (B) *The z1A2 locus is the youngest cluster among the 19-kDa zeins*. Created as a result of a translocation from the *z1A1* locus on chromosome *4S* it is populated by only 3 zeins, the second one being seeded here on top of an inactive helitron. (C) *The z1B locus is almost identical between the two inbreds*. With its 9 zein copies, it is positioned on chromosome *7S*, and except for a 1 kb indel the two inbreds are almost identical over more than 260 kb. (D) *Massive expansion of intergenic regions at the z1D locus*. Present on *1S*, this locus contains 5 zeins.

Another Stiff Salk Synthetic line that is of great interest is BSSS53 because of its high methionine content [20]. Because both lines are derived from the same breeding experiment, one could arguable use them as a model for haplotype variability of common inbreds. We used two BAC libraries (see Materials and Methods) of this inbred to isolate the complete set of α-zein genes. Because allelic gene copies are more conserved than tandemly duplicated copies, each chromosomal region of the two inbred lines was aligned with conserved sequences to illustrate sequence variability (Figure 2). In total, 41 α-zein genes, in B73, and 48 in BSSS53, are positioned on three chromosomes (*1S*, *4S*, and *7S*) and form five distinct loci (three on chromosome *4S*).

### Variability of gene copy number between different inbred lines

Differences in gene copy number seem not to be a general feature for all α-zein loci. Besides the *z1C1* cluster the *z1A1* cluster is the only other one that varies in gene copy number, both being physically linked to the *z1C1* by a 300 kb segment. Three overlapping clones of BSSS53 were aligned to the *z1A1* B73 allelic region (Figure 2A) (accession GQ214222.1). The *z1A1* locus differed by only one copy, with 8 in BSSS53 and 9 in B73. Taken together, within about half a megabase, BSSS53 has 30 α-zeins and B73 has 23, but haplotype variability and gene content is very uneven over the entire length. The degree of variability is best illustrated by the differential expansion of these closely linked regions. Deletion or insertion of gene copies is more dramatic in the *z1C1* region than in the *z1A1* region and its gene density is higher (Table 1). The allelic regions of the *z1C1* cluster differ in size between 169 kb in BSSS53 and 111 kb in B73, while the allelic regions of the *z1A1* cluster are nearly the same with 107 and 104 kb, respectively.

**Table 1:** Haplotype variability at the α-zein gene loci.

	Inbred	Conserved (%)	REs (%)	TEs (%)	Genic (%)	Intergenic (%)
***z1A1***	B73 (104 kb) BSSS53 (107 kb)	83 81	35 52	10 10	6 5	49 33
***z1A2***	B73 (104 kb) BSSS53 (107 kb)	91 96	47 42	21 22	3 3	31 33
***z1B***	B73 (104 kb) BSSS53 (107 kb)	99.5 100	51 51	3 3	3 3	44 43
***z1C2***	B73 (104 kb) BSSS53 (107 kb)	75 50	14 11	4 5	9 9	73 76
***z1D***	B73/BSSS53*	99	n/a	n/a	n/a	n/a
*z1D*	B73 (104 kb) BSSS53 (107 kb)	58 58	62 50	7 9	1 1	30 40

*single copy

We used nucleotide synonymous substitution rates (Ks values) to determine the chronology of tandem amplification events. The first *z1C1* and *z1A1* genes arose before the split of the *Andropogoneae* tribe 11.9 mya, probably by the insertion of one gene copy followed by unequal crossing over within the coding region because the resulting two tandem gene copies encode a 19-and 22-kDa zein with different number of internal repeats. These two new gene copies each seeded tandem clusters now separated by 300 kb of chromosomal DNA containing non-related genes and transposable elements (TEs) [9]. As a result the *z1A1* region has mostly 19-kDa zein and the *z1C1* region mostly 22-kDa zein genes. When the two allelic *z1A1* regions are aligned, the only non-allelic 19-kDa zein gene present in B73 arose about 0.5 mya. This event, however, did not result from unequal crossing over within the coding region, but is rather a tandem duplication of ∼5 kb (Figure 2A). Another tandem duplication resulted into the *z1A1-3* and *z1A1-4* copies also about 0.5 mya, present in both inbreds.

### Conservation of gene copies in B73 and BSSS73

It is not surprising that older copies became damaged because the newer ones could assume the role of providing storage proteins for the seed. The older copies at the *z1A1* locus (i.e. *z1A1-1*, *-8*, and *-9*) are either severely truncated at the 3′ end (*z1A1-9* is only 332 bp long), missing the A from the start codon (*z1A1-8*) or have a premature stop codon (*z1A1-1*) in both inbred lines (Figure 2A) (phylogenetic trees constructed for each locus are available as Figure S1).

The *z1A2* locus (Figure 2B) (BSSS53 accessions GQ214223.1 and GQ214224.1), which arose 2.2 mya after allotetraploidization (Table S1) is populated by three zein gene copies, in both haplotypes, spread over 120 kb in B73. In BSSS53, we isolated two BAC clones that provided us with the genomic sequence of the three zein genes, but it proved to be difficult to design a PCR probe based on the large fragment between *z1A2-2* and *z1A2-3*, which is mainly composed of retroelements (REs). Interestingly, the oldest copy for this locus, *z1A2-2*, appears to be present within a helitron element, characterized by the 5′-TC, CTAG-3′, hairpin sequences upstream of 3′ end, and a host nucleotide that is a G instead of the regular A. Because it lacks a gene encoding the helicase, it would be classified as a non-autonomous element [21]. No other genes besides the zein are present in the helitron. To determine whether the helitron has copied the zein gene, we screened 32 inbred lines. However, there appear to be no haplotypes lacking the *z1A2* locus, different to the cytosine deaminase gene, for example, linked to *z1C1* locus that was also a paralogous copy in some inbred lines but not others [22]. This might suggest that the *z1A2* insertion occurred after and independent of the helitron movement. The other two zein copies at this locus are either intact (*z1A2-1*) or have a premature stop codon (*z1A2-3*).

The *z1B* locus has nine tandem copies that are spread over ∼200 kb. It took three overlapping BACs from BSSS53 (accession GQ214225.1) with a total length of more than 260 kb to cover the allelic complement of the B73 *z1B* locus (Figure 2C). Over a length of more than 260 kb, except for a 1 kb indel, the two haplotypes are nearly identical, with more than 97% sequence homology. This is twice the length of the *z1A1* locus for the same number of zein genes, illustrating a rather large expansion of intergenic space. The original zein gene copy that inserted before allotetraploidization is *z1B3*. Through subsequent copying events the other eight copies were generated. Six of them do not have additional insertions between them, one example being the *z1B1* and *z1B2* genes. Another is the duplication of a pair resulting in *z1B4*, *-5*, *-6* and *-7*. It is interesting that the pair *z1B4* and *z1B6* that arose from the most recent amplification (Figure S1) appears to be intact. All the other seven copies have accumulated premature stop codons but none of them is truncated.

The *z1D* locus is characterized by a massive expansion of intergenic regions (Figure 2D). In B73, three overlapping clones generated a total of ∼480 kb contiguous chromosomal sequence, where the five zein gene copies are spread over more than 300 kb [19]. Overlaps, however, had to take advantage of the deep coverage of the B73 BAC libraries (30x), which were not available for BSSS53 (3.5x). Indeed, the low gene-density in this region made it impossible to isolate a complete allelic complement from BSSS53. However, two BSSS53 BAC clones containing all the *z1D* genes were isolated and sequenced (accessions GQ214226.1, and GQ214227.1). Both clones were aligned with the B73 sequence based on the zein gene copies and TEs that inserted into this region before different haplotypes emerged. The most recently amplified zein copies, *z1D2* and *z1D4* (Table S1), are intact, while all other copies are damaged. They have either been truncated like the *z1D1* gene or accumulated stop codons like the *z1D3* and *z1D5* gene copies. In addition to the stop codons, these two copies acquired RE insertions in B73. The insertion in the latter copy occurred as recently as 0.12 mya, while the first one has its reading frame disrupted by a solo LTR.

### Haplotype divergence based on retrotransposition

The major force of DNA mobility in the maize genome has been retrotransposition of LTR retrotransposons. When a retrotranscript inserts into a chromosomal region, it generates LTRs, which are identical at the time of insertion. One can assess the relative times of RE insertion events in each chromosomal region based on the Ks values of LTRs, which is two fold higher than that of a gene [23]. Such an approach is very helpful to gain insights into how these insertions relate to the insertion of gene copies. For instance, does the *z1D* locus harbor the oldest REs and the *z1A2* the newest, respectively, according to the young age that the genes in this region have?

We looked at REs that are shared between the two inbreds, others that are specific to one or the other and also the nested ones, with a subcategory for nested elements that are also haplotype-specific (Table S2). One can immediately notice the contrast between the *z1A2* and the *z1D* loci. The latter one is the site for very old insertions, some that even precede the allotetraploidization event: the *Gypsy35* element that is 6.2 million years old being a good example (Figure 2D*). Insertions like this are a good indicator that the two progenitors of maize had already undergone some retrotranspositions of the same elements, a phenomenon that became so active after allotetraploidization. Although the *z1D* locus is characterized by other insertions as old as 4 mya (not found in any other loci), it is still prone to acquiring additional insertions as shown by the nested haplotype-specific *Gypsy73* (Figure 2D**) element, whose LTRs are identical. In fact, the *z1D5* zein gene in B73 has another one of these recent haplotype specific insertions, with a *Zeon2* element (Figure 2D***) inserting 0.12 mya, as described above. On the other hand, the *z1A2* locus has no REs older than 1.5 mya, no nesting and a very recent haplotype specific insertion: the *Zeon2*, with both LTRs identical (Figure 2B* and Table S2). In general, haplotype specific insertions are younger than 2 mya, with the oldest ones present at the *z1C* and *z1D* loci. Also, the age of the nested REs among all loci is less than 1 mya, with the exception of *z1D* locus with two REs that are close to 2 mya. It is here that the biggest cluster of nested elements was identified having about 60 kb in size. Nesting is almost absent for the *z1A1* and *z1A2* loci, with only one insertion (*Zeon2*; 1.19 mya) in the oldest RE (*Prem2*; 2.04 mya) (Figure 2A* and **, respectively) of the first locus.

### Allelic gene expression of the 19-kDa zein genes

An interesting question that arises is whether haplotype divergence might have an impact on gene expression. We previously used abundant EST resources to determine which genes are expressed [9]. However, these resources did not provide comparable quantitative levels and did not include BSSS53. To determine which gene copy is expressed, at what level, in which inbred, we created cDNA libraries from immature endosperm at 18 days after pollination (DAP) from B73 and BSSS53 and their reciprocal crosses. We designed three universal primer pairs to amplify nearly full-length zein sequences specifically for the *z1A1* and *z1A2* loci, *z1B* and *z1D*, and *z1C* loci, respectively, and then randomly sequenced several 96-well plates (enough to detect a zein gene expressed at a threshold of 0.3%) for each sample and compared the results with genomic sequences.

**Figure 3:**
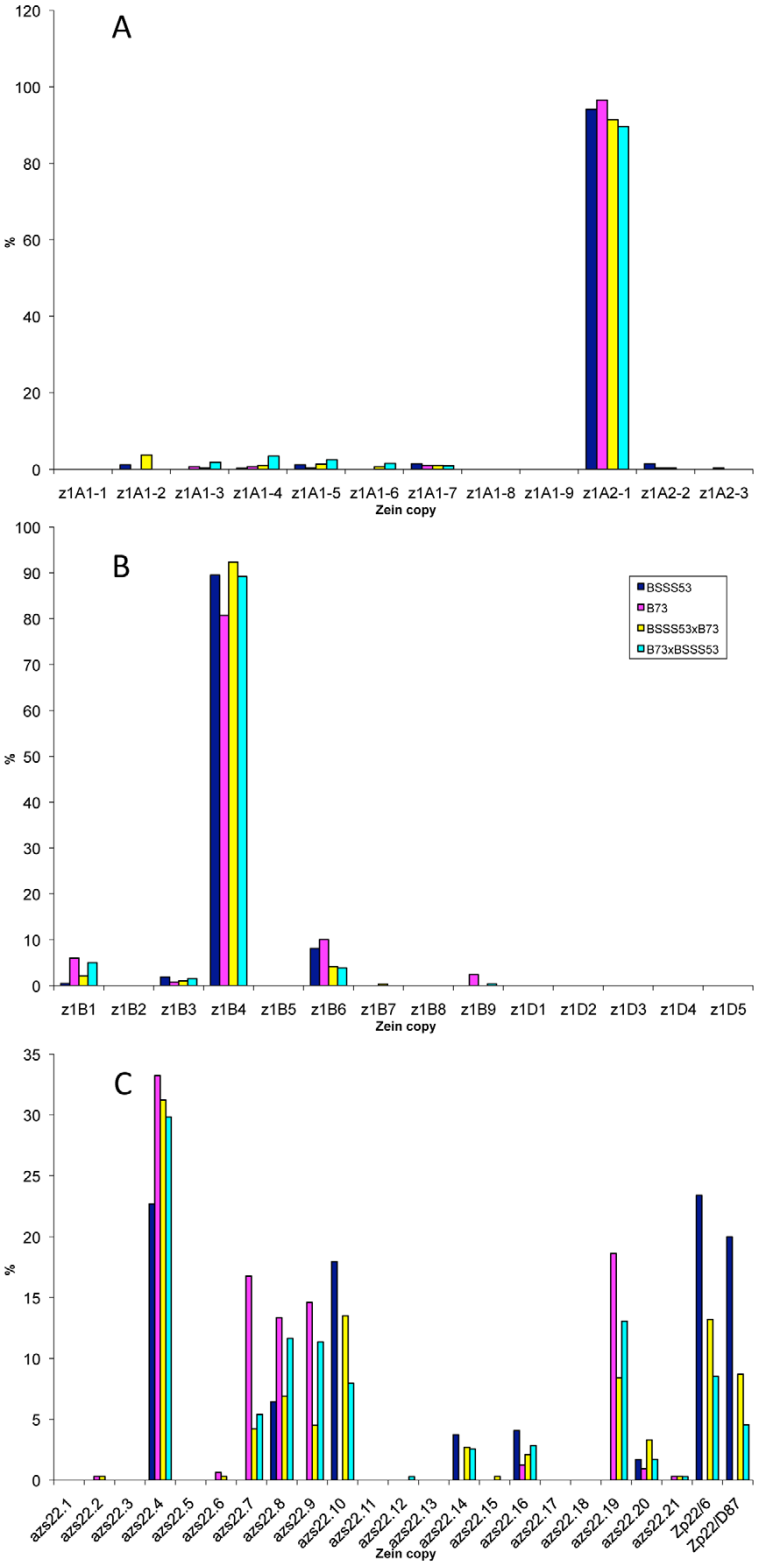
Transcript levels of the youngest zein gene copies prevail at each of the loci analyzed in B73, BSSS53, and their reciprocal crosses. Expression levels at the two *z1A* loci (A), *z1B* and *z1D* (B) and *z1C* (C), respectively, using immature endosperm harvested 18 DAP. Each zein copy is represented on the *x-axis* and its percentage out of the total transcript pool on the *y-*axis.

It is quite striking that in the case of the four 19-kDa zein gene clusters only two out of 26 gene copies are expressed at high levels (Figure 3A and 3B). They are the same copies in both inbreds with no quantitative differences of expression in reciprocal crosses. Moreover, based on Ks values, these two copies would represent the most recently amplified gene copies. That is not say the older gene copies are not expressed, but at very reduced levels or not at all. In case of the two *z1A* loci a single gene copy, the transcripts of *z1A2-1*, account for more than 90% of the total pool (Figure 3A). This result differs from a recent expression study of α-zein genes [24]. Although the study also shows that *z1A2-1* has the highest level of expression among the z1A gene copies, it seems to exhibit less specificity for individual copies. Indeed, primer selection for PCR seems to be the critical difference in respect to the length of primers, mismatches of primers to different clusters, and length of PCR products covering polymorphisms. Our study also used deeper sequencing of samples and was done for two inbreds with known genomic sequences and their reciprocal crosses. On the other hand, it was important to see that developmental expression does not seem to switch the relative contribution of each gene copy [24], which permitted us to simplify our study by sampling a single developmental time point. The *z1A2* locus is derived from *z1A1* (paralogous) and *z1A2-1* copy is the most recent tandem amplification, having the same age as *z1A2-3*, 1.4 mya (Table S1). Despite an in-frame stop codon, the latter one is expressed although to very low levels at least in B73. There is one non-allelic gene copy, *z1A1-6*+B73, that is absent in BSSS53; it is expressed in B73 and in its hybrid with BSSS53 although at very low levels.

The other 19-kDa zein gene clusters - *z1B* and *z1D* - also have only one gene copy expressed at high levels, which is again the most recent tandem amplification, *z1B4*, accounting for more than 80% of the total transcripts (Figure 3B). It together with *z1B6* are the only ones that have an intact ORF, while the others have all accumulated in-frame stop codons, and are expressed at low levels, if expressed at all. Another inbred that we have analyzed (W22) has a slightly different pattern of gene expression with the *z1B1* transcripts ranking second after *z1B4*, with more than 20% of total (not shown). Analyzing its sequence we found that it has an intact ORF, unlike the alleles of B73 and BSSS53, indicating allelic variations of stop codons as shown previously for *z1C1* gene copies [13]. The *z1D* zein genes appear to be silenced, with only two copies (*z1D2* and *-4*) having intact ORFs; the others have either been truncated, accumulated stop codons, or had REs inserted on top of them.

### Epigenetic resetting of the α-zeins in endosperm tissue culture

**Figure 4:**
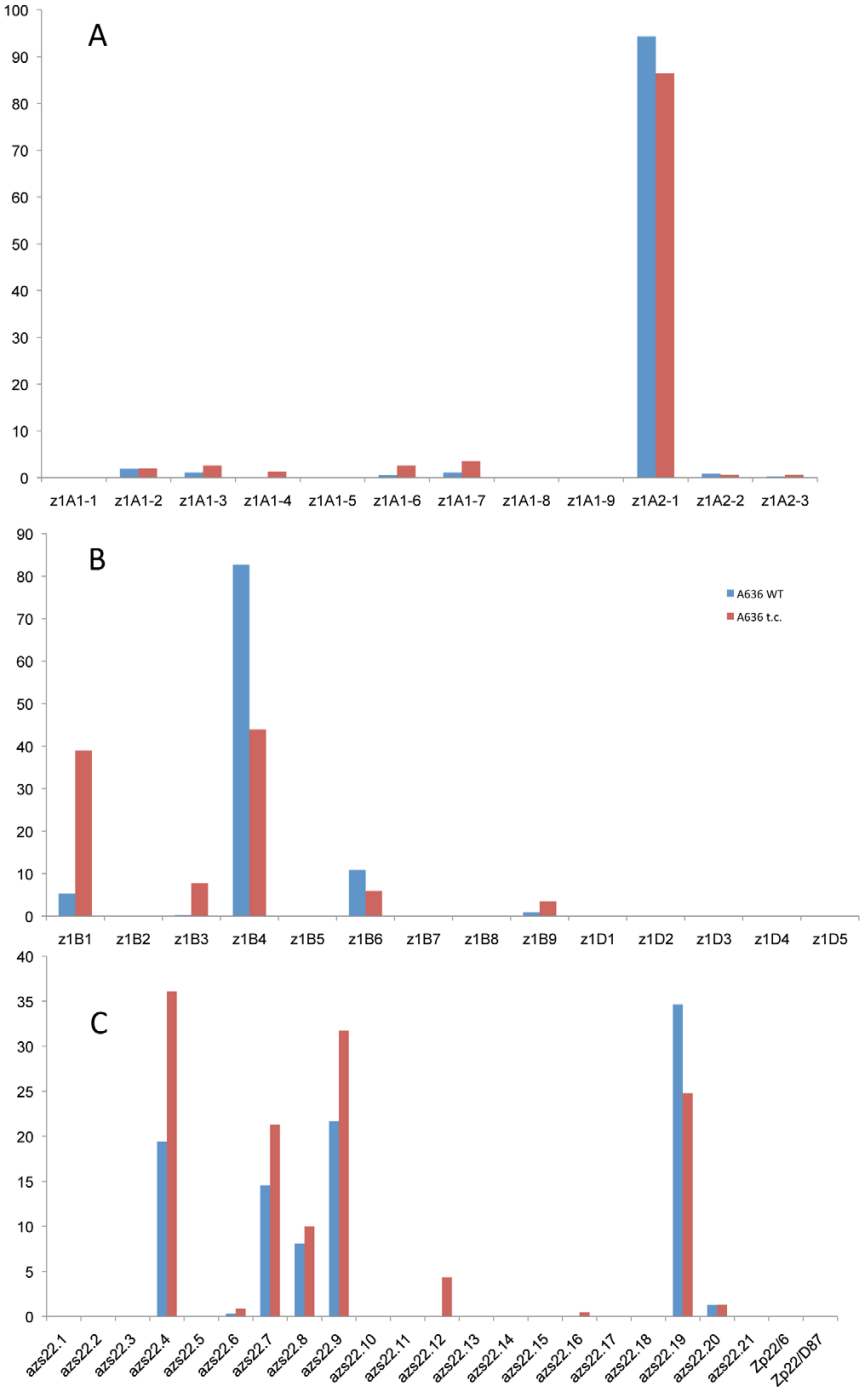
Tissue culture induces epigenetic resetting of the α-zeins genes expression in A636 inbred. While the *z1A* loci show little effect under tissue culture conditions (A), the *z1B* and *z1D* (B), and *z1C* (C) loci undergo major shifts in gene expression, with older copies getting reactivated or having a boost in their activity and younger ones contributing less to the total transcript pool.

Given the expression potential of gene copies and the variability within the same gene family, we investigated whether gene expression could be changed by induction. A simple device for doing so is to culture differentiated cells. Indeed, tissue cultures have been shown to be responsible for turning on genes that are normally silent *in vivo*. For example *Tos17* retrotransposon is activated in tissue culture of rice and this is due to cytosine demethylation [25]. It also has been shown that demethylation occurs at high frequency in tissue cultures of maize. Therefore, demethylation has been proposed as the main source of tissue culture-induced variation [26]. Previously, it was shown that one specific inbred, A636, can be used to initiate maize endosperm cultures that faithfully maintain expression of storage protein genes [27]. A new culture was initiated as described previously and A636 endosperm was cultured for several weeks as described under Materials and Methods. RNA was then isolated from the callus cultured on solid and liquid media and cDNA libraries were created from RNA of A636 immature endosperm and A636 cultured endosperm cells. Sequencing of random cDNAs followed the same protocol as for the B73 and BSSS53 inbreds. The sequences generated were then compared to the genomic sequences of those two. There is clearly a difference in the expression pattern between normal and cultured endosperm (Figure 4). For some gene copies, expression appears to be induced like *z1B3* and *azs22.12*. While *z1B3* has a premature stop codon, *azs22.12* is a complete and intact gene copy in A636, B73, and BSSS53 that arose very recently (0.6 mya). Therefore, *azs22.12* represents an example of a gene copy that was reactivated through the tissue culture process. The more common changes are expression levels. Expression is reduced for *z1B4* and *azs22.19*, but enhanced for *z1B1*, *azs22.4*, *azs22.7*, and *azs22.9*. In contrast to the *z1C* and *z1B* loci, *z1A* loci do not seem to be significantly affected under tissue culture conditions (Figure 4A). Genes at the *z1D* locus remain silent, although two of them have intact ORFs. On the other hand, presence of a premature stop codon in the gene's ORF does not prevent enhanced expression after tissue culture treatment. For example *z1B1* has an in-frame stop codon but is expressed at higher levels under tissue culture conditions. On the other hand, genes that have intact coding regions can be down regulated, like it is the case for *z1B4* and *z1B6*, for example. Therefore, changes in the expression levels of *trans*-acting factors through tissue culture might also play a role in quantitative levels of expression.

### Divergence of promoter regions

Because even genes with premature stop codons are still transcribed, it appears that selection for conserved gene sequences also extends to the promoter regions of the α-zein genes. Although little is known about specific transcriptional activators of α-zein genes, they share a sequence motif with many other storage protein genes, GTGTAAAG, which occurs about 300 bp upstream of the translation start site and is called the −300 element or the P-box (prolamine-box) [28]. This element acts as an enhancer in a transient expression system and binds to the prolamine-binding factor (PBF), which has been identified as a maize domestication locus [29], [30], [31]. A second trans-acting factor that is known is *opaque2* (*o2*). However, in *o2* mutants some α-zein gene copies are still expressed, indicating a redundant system of factors. We therefore compared the upstream region of all α-zein gene copies to identify sequence motifs within a window of 500 bp that might deviate from a consensus sequence using the PLACE database [32]. Indeed, functional genes have the P-box core motif and some of the non-expressed genes have mutations in this motif (Figure S2 and Table S1), consistent with the role of PBF as a regulator of zein gene transcription. Besides the P-box, we can also find a sequence motif for the *o2* transcriptional activator (Table S1). It is present 171 bp upstream of the start codon, on the lower strand, for the *z1A* loci, 181 bp for the *z1B* locus, and 178 bp for the *z1D* locus.

### Methylation patterns of the α-zein genes promoters

**Figure 5:**
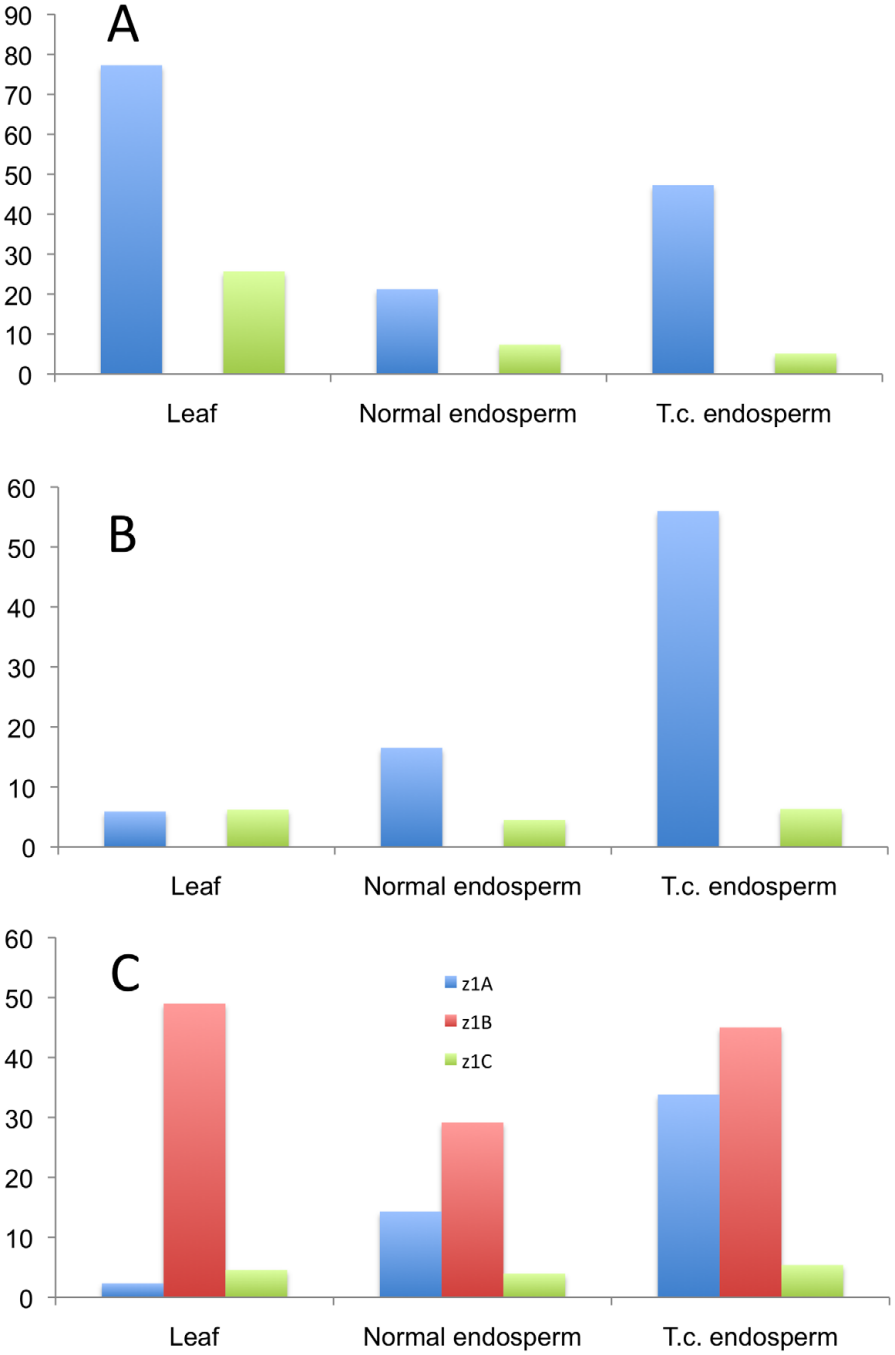
Methylation pattern in the promoters of three α-zein genes loci. The three loci are analyzed in their (A) CG-, (B) CHG-, and (C) CHH-contexts. The bars represent averages of methylation percentages corresponding to individual cytosines present in the 500 bp promoter sequence (shown in detail in Figure S4).

Because the methylation status of the promoters plays an equally important role in gene regulation, along with the presence of binding sites for various transcription factors, we analyzed the three expressed loci (*z1A*, *z1B*, and *z1C*) by bisulfite-sequencing 500 bp upstream of the start codon in three different tissues: normal and tissue-cultured endosperm, and leaf. Due to the high sequence homology in the promoters of paralogous gene copies it would be virtually impossible to analyze individual promoter sequences. Therefore we used universal primers for the three loci mentioned above to get an insight into their cytosine methylation status. The differences are striking and hint towards a possible different gene regulation mechanism that is locus-specific (Figure 5 and Figure S4). The *z1A* locus is characterized by five highly methylated cytosines, in leaf tissue (Figure S4A, black arrows), whereas the rest of the promoter maintains a very low methylation level. The pattern is very similar to that of the *z1C* promoters, where there are four highly methylated cytosines, one of them representing the binding site for O2 (Figure S4C; black and dark purple arrows, respectively). All five peaks at the *z1A* loci are in CG context, whereas only the one mentioned above for the *z1C* loci is in CG context, among the four. Very basal methylation is present along all the other cytosines in leaf DNA. Surprisingly, the endosperm grown under tissue-culture conditions has higher methylation levels than its normal counterpart, for *z1A*. This is not the case for *z1B*, where it behaves as an average between high and low peaks detected in leaf and normal endosperm, a unique pattern characteristic to this locus. Another striking difference is the high methylation patterns of the *z1A* and *z1B* loci in endosperm, when compared to *z1C*, all cytosines being less than 10% methylated here; another possible indication of the different transcriptional regulation of the 19- versus the 22-kDa zeins. Furthermore, the overall methylation of the *z1C* promoters is lower in all contexts and tissues analyzed. Interestingly, all cytosines at the *z1B* locus are in CHH context only, while the other two loci are characterized by CG, CHG and CHH methylation.

## Discussion

An unexpected result in the analysis of plant genomes has been that haplotype variability extends beyond nucleotide polymorphism to large-scale insertions and deletions, including genes, and could extend up to 2.6 Mb segments that are present in one but not the other haplotype [33]. Here, we investigated how this variability extends throughout the entire α-zein gene subfamily, which spreads over three of the ten maize chromosomes in six distinct locations. Our study gives an in-depth view of haplotype variability at the level of a specific medium-size gene family in maize rather than global [33] or single loci [34]. Because each location of the α-zeins, except for the *z1C2* locus, contains tandem arrays of gene copies, they occupy large chromosomal regions. The largest region comprises two loci, *z1C1* and *z1A1*, about 540 kb in size on B73 chromosome *4S*. We do not know the size in BSSS53 because of the lack of a fingerprinted map, but each cluster by itself is quite variable in size. While the *z1A1* region is about 100 kb for each haplotype, the *z1C1* region is 111 in B73 and 169 kb in BSSS53. The second largest is the *z1D* region with 300 kb, followed by the *z1B* region with 200 kb, and the *z1A2* with 120 kb in B73. Although expansion of intergenic regions by retrotransposition is common to all zein gene loci, it seems to have been most active at the *z1D* locus. Although retrotransposition occurred before allotetraploidization, it was rather infrequent compared to recent times. Based on comparison with sorghum, maize had a greater activity of transposition, with additional copying of prolamine genes in tandem and also to unlinked positions as exemplified by the *z1A2* and *z1C2* loci on chromosome *4S* and *z1B* on *7S*, respectively. Diploidization possibly set in motion further divergence of homoeologous regions of maize as we can see from the *z1B* locus. Although this locus formed already before allotetraploidization, most retrotranspositions occurred between 0.1–3 mya. Interestingly, Ks values vary for common insertion events albeit not drastically. This variation indicates that haplotypes differ in Ks values if no apparent selection applies. However, it seems to be more parsimonious to suggest that rates changed after establishing different haplotypes because of a change in recombination rates, which could counteract nucleotide substitutions by sequence conversion. Consistent with this assumption is that haplotype variability occurred more recently. For instance, 52% of the *z1A1* cluster in BSSS53 is composed of REs, but in B73 only 35%. The difference is due to elements that inserted only recently in one of the two haplotypes. We can clearly see that after chromosome expansion, additional retrotransposition resulted into segregating genotypes that remained stable. These genotypes constitute haplotypes that mainly differ in the intergenic space of these gene clusters. Interestingly, the percentage of TEs at all α-loci is significantly lower than the maize genome average, estimated at almost 80% for Maize 4a.53 release. Although we can find examples of REs that inserted into zein gene copies (e.g. *z1D3* and *z1D5* in B73) the reverse is not true despite that some zein genes were copied very recently. However, insertion into a gene might be favored if it is already damaged because both *z1D* genes had already accumulated stop codons in both inbred lines. While the nesting effect is also very recent it does not extend to zein gene copies to insert into other zein genes.

Although we can observe some variability in the copy number of zein genes, particularly in the *z1C1*-*z1A1* region, it is surprising how low this is compared to other gene clusters like the *rp1* locus on chromosome 10 [35]. For instance, unequal crossing over could result in a change of gene copies. However, reconstruction of all gene clusters indicates a different mechanism of gene amplification. Although it is tempting to speculate that because of chromatin structure recombination would occur within actively expressed copies, we actually do not know whether recombination occurs preferentially within certain copies of a gene cluster. One would even expect gene conversion to reduce allelic diversity. However, chromosome alignments would have to be quite precise because of selection against unequal crossover between two conserved REs, which otherwise would lead to loss of gene copies.

One interesting feature, common to all α-zein genes is that the most recently amplified gene copies contribute the most to mRNA accumulation. The older copies either accumulated premature stop codons or are truncated (Table S1). Presumably, older gene copies accumulate more mutations, gene truncation, even gene loss, and chromosomal rearrangement because the younger ones can complement a loss of function [36], [37], [38], [39]. In the case of the 22-kDa zeins, the younger zein copies (*Zp22*/*6* and *Zp22*/*D87*), which arose by a segmental duplication, are responsible for nearly 40% of the total *z1C* transcripts and this causes a shift to 65% of the transcripts being attributed to the new genes [11]. The transcriptional regulator O2 was no longer regulating their expression, which is true for the other α-zein gene loci and possibly could be explained by the interaction of other transcription factors with DNA binding motifs in the upstream promoter regions. Here, we also can show that, based on the phylogenetic data, the youngest zein gene copies are the ones that accumulate mRNA to detectable levels while the older ones have either accumulated stop codons or have been truncated.

The methylation status of the promoters and the gene bodies themselves also seem to play an important role in the regulation of this family. Gene bodies of storage protein genes have been shown to be undermethylated in endosperm when compared to different somatic tissues and embryo, where a common methylation pattern was reported [40]. A more recent study corroborates the undermethylation in gene bodies with a CG depletion of duplicated sequences and speculates that the higher the expression of a gene is, the more CG depleted its sequence will be [41]. Our study confirms the observations but also extends the analysis to promoters of the different 19-kDa zein loci. In addition, we could show that other cytosines than the one inside the ACGT core sequence of the promoter of the 22-kDa zeins are highly methylated in the leaf and not in the endosperm. These could potentially play a role in the regulation of the newer 22-kDa zein copies that are not under the control of O2 [11].

Analysis of expression levels for members of the α-zein gene family has provided us with evidence that indeed the epigenetic state of each copy is an important factor in reviving older copies from a silenced state. We hypothesize that the longer the endosperm cells are cultured in liquid media the more likely it is that gene copies will return to their original methylation state (Figure S3). It is interesting to note the difference between gene copies at the *z1A* loci and the rest of the α-zeins. Whereas all the others show obvious effects on the expression levels when grown under tissue culture conditions, these genes are less affected. This would suggest that they might be under control of different regulators than the rest of the family members. Just like the O2 transcription factor does not extend its influence over younger zein copies at the *z1C1* locus (*Zp22*/*6* and *Zp22*/*D87*) [11] the gene copies at the *z1A2* locus, which arose after allotetraploidization from a translocation event originating at the *z1A1* locus, might be under the control of different transcription factors. It is also interesting to note that among the 19-kDa zeins, the *z1A* copies are the only ones that have the position of both the P-box and the Opaque2ZMB32 motifs shifted by 10 bp, closer to the start codon (Table S2).

Each of the loci analyzed for their methylation patterns in the promoter region differs from one another, probably due to position-specific influence caused from insertion into unlinked locations in respect to the donor copies. A recent study in rice endosperm shows that methylation is lower in all sequence contexts with a drastic decrease in CG methylation (93% of embryo level), 2x decrease for CHG and 5x decrease for CHH contexts [42]. This does not seem to be the case for maize endosperm, with fluctuations unique to each of the loci analyzed. For example, the *z1B* promoters are characterized by neither CG nor CHG contexts. Only CHH methylation is possible and the pattern is distinct, but overall having a decrease in methylation of the endosperm, compared to leaf. The *z1C* promoters, on the other hand, show significant drops in CHH methylation, not just CG or CHG, whereas *z1A* is characterized by significant decreases in CG methylation, as observed for the five highly methylated cytosines, but overall higher methylation for the others. Therefore, differential methylation patterns could be a position effect and result in differential expression of members of a multigene family.

## Materials and Methods

### Plant material

B73 and BSSS53 plant material came from our lab stocks while A636 seeds were a gift from Dr. Hugo K. Dooner at the Waksman Institute.

### BAC library construction and screening in BSSS53

Initial screening of the BAC library already available in BSSS53 [15] was done by PCR with degenerated primers based on zein gene sequences. Once a BAC pool was identified as positive, it went through several rounds of dilutions until a single colony was identified and grown on LB plates. We later switched to screening the pooled BACs by filter hybridization. New primers were developed, that were specific for each of the zein loci and they were used to screen the BAC pools by PCR. Once a pool was identified as positive we diluted an aliquot of the stock in LB medium and directly streaked it on LB agar plates. Single colonies were then picked and grown on filters that were later hybridized with a PCR-generated probe specific for each locus. Due to the low coverage of the existing BAC library, a new one was created in order to isolate the BAC that contains the first two zein copies at the *z1A2* locus. We partially digested genomic DNA with *Hind*III enzyme and cloned the fragments into the pINDIGO-BAC-5 vector from Epicentre. Both libraries combined had an average insert size of 100 kb and a genome coverage of 4.5x. All positive BACs were sequenced in a 3730xl DNA sequencer using the BigDye terminator chemistry (Applied Biosystems) by “shotgun” strategy up to 8x coverage.

### Sequence assembly and annotation

BAC sequences were assembled using PhredPhrap and then went through a first round of annotation using a series of software available on-line: BLAST suite from NCBI was used for homology searches and sequence comparison between the two inbreds, RepeatMasker for TE searches, and SoftBerry for gene prediction models. Sequences were then manually annotated, false gene predictions were eliminated, TSDs (target site duplications) and LTRs were identified for the TEs, and then the two haplotypes were aligned. A threshold of at least 95% homology was set when comparing the two sequences.

### LTR analysis

To estimate the insertion time for the REs we used the left and right LTRs sequences that we input in the Mega4 software to calculate the nucleotide synonymous substitution rates (Ks values). Default settings were changed to Distance and Std. Err., Pairwise deletions and Kimura 2-parameter. We then used the Ks value reported for LTRs [23] to calculate the insertion time. To avoid any bias, we removed any indels or sequencing gaps from the LTRs before comparing their sequence.

### Expression analysis

For the cDNA analysis in B73 and BSSS53 we used immature endosperm tissue harvested 18 DAP. RNA was extracted using the Spectrum Plant Total RNA Kit from Sigma, which was reverse-transcribed using the SuperScript III First Strand Synthesis Kit from Invitrogen. The cDNA was PCR-amplified with three primer pairs: one for the *z1A* loci (5′ primer: CTCTTAa/gATTAGTAGCTAATAt/cATC; 3′ primer: CTGGGAAGCCACAAACATCA), one for *z1B* and *z1D* loci together (5′ primer: ATTAGTCGGTAATCCATCAACC; 3′ primer: CTAGAAGATGGCACCACCAATG), and one primer pair that had been previously used for the 22 kDa zeins [11]. PCR products were ligated in the pGEM-TEasy vector from Promega and then transformed into *E*.*coli* cells (ElectroMAX, DH10B; Invitrogen). We then randomly sequenced cDNA clones with universal primers from both ends. The consensus sequence of the two reads was obtained using SeqMan software (part of DNASTAR Lasergene package). The consensus sequences were then blasted against a database containing the genomic sequences of all the zein gene copies from the two inbreds, and the hit with the highest score was recorded. All the hits for each individual gene were summed and the value converted in percent of total transcripts.

The tissue culture experiment followed the same steps as above with the only difference that 13 DAP immature endosperm was used instead and the inbred line was A636. The endosperm harvested from the same ear that was used to isolate RNA for immediate analysis of transcript levels *in vivo*, was used to induce the tissue culture. Same culture medium and conditions were applied as in [27]. One month later callus was regenerated from the endosperm. This was transplanted on a fresh solid medium and grown for two more weeks and then used to isolate RNA. After that, the tissue culture was maintained in liquid media, having the same composition as the solid one, minus the agar. A new batch of fresh callus was collected after two months of sub-culturing and used to isolate RNA, which was later used in the analysis presented as Figure S3.

### Promoter sequence analysis

500 bp upstream of the start codon were used to search for motifs that are either shared or unique in all the members of the α-zein gene family. The search was done using PLACE [32].

### Bisulfite sequencing

Genomic DNA of A636 from normal endosperm, tissue culture-grown endosperm and leaf tissue was treated according to the protocol of Epitect Bisulfite Kit from Qiagen, for bisulfite conversion. Universal primers were manually designed for amplifying the z1C zein copies (5′ primer: ACATGTGTAAAGGTGAAGAG; 3′ primer: GGTCATTACTAATACACTTCAC). For the z1A and z1B clusters regions of high conservation among all zeins at the specific cluster were analyzed and then primers were designed in those regions using the z1A2-1 and z1B4 promoters as reference, respectively. Methyl Primer Express Software, freely available from Applied Biosystems, was used for the design. z1A 5′ primer: AGTGATTTTTTAAATYGATTATTAT, z1A 3′ primer: TATTTATACACATATCAATCCTTATACTT. z1B 5′ primer: TATGTGGTTAATGTTATATATGTGTAA, z1B 3′ primer: TTATTACTACTAAATTCCACTTTCTATATT. After PCR amplification the products were cloned into pGEM-TEasy vector from Promega and one 96 well plate was sequenced for each of the samples; i.e., one for leaf DNA, one for normal endosperm and one for tissue culture-generated endosperm, with each of the three primer combinations, respectively. The consensus was obtained using SeqMan. Then the sequence was scanned for all the cytosines, to look for site of conversion.

## Acknowledgments

We would like to thank Moisés Cortéz-Cruz and Amy Nelson for initial BAC library screening and sequencing in BSSS53 and Galina Fuks and Rémy Bruggmann for their help with the bioinformatics analysis. We also appreciate advice and help from members of the Dooner Lab at Waksman Institute.

## Supporting Information

Figure S1 NJ and UPGMA trees constructed for each of the 19-kDa zein genes loci. At each locus an NJ tree was constructed for both inbred lines, followed by a UPGMA tree and then the same two methods were implied to construct a common tree with zein copies of both inbreds. We chose sequences of the prolamine genes in sorghum (kafirins) as outgroup. Genomic sequences were aligned using ClustalW and then Mega4 software was used to generate the trees. Bootstrap values are indicated on the branches of the tree, for 1,000 replicas.
(PDF)

Figure S2 P-box conservation in the promoter region of the 19 kDa α-zeins in both inbreds. Red letters indicate SNPs in the P-box sequence of several zein genes.
(PDF)

Figure S3 Extensive liquid tissue culture tends to re-establish the original methylation state. This is mainly visible for expression levels of copies at the *z1C* locus (C). Copies at the *z1AI* (A) and *z1B/D* (B) loci maintain the same pattern as in the original stages of tissue culture.
(PDF)

Figure S4 Methylation pattern in the promoters of *z1A* (A), *z1B* (B) and *z1C* (C). The positions of the cytosines present in the consensus sequence of each locus are marked relative to the start codon. Red label – CG methylation; Black label – CHG methylation; all the other data points are CHH methylation.
(PDF)

Table S1 A summary with the status of the zein genes, the position of the P-box, and O2 motif in the two inbred lines as well as the estimated age of each of the zein copy.
(XLS)

Table S2 Insertion times of REs. Each element at each locus was assigned to one of the 4 categories color-coded in the legend, based on its sequence context. Using Ks values for the left and right LTRs we estimated the age of insertions in mya +/− the standard deviation.
(XLS)
