# Supplementary figures and images for: Differential Gene Expression and Epiregulation of Alpha Zein Gene Copies in Maize Haplotypes
Source: PLoS Genet. 2011 Jun 23;7(6):e1002131. doi: 10.1371/journal.pgen.1002131 (PMC3121756; doi:10.1371/journal.pgen.1002131)

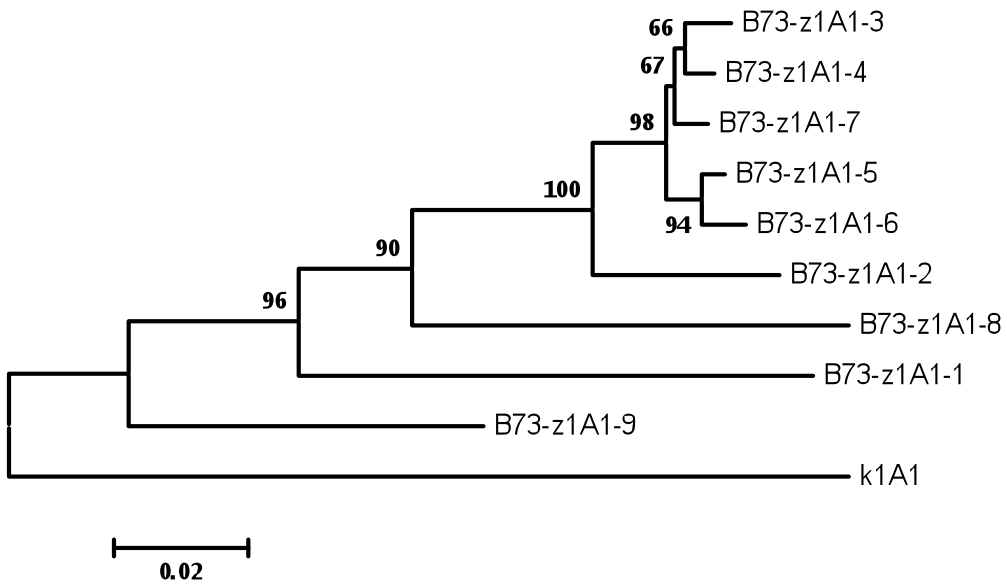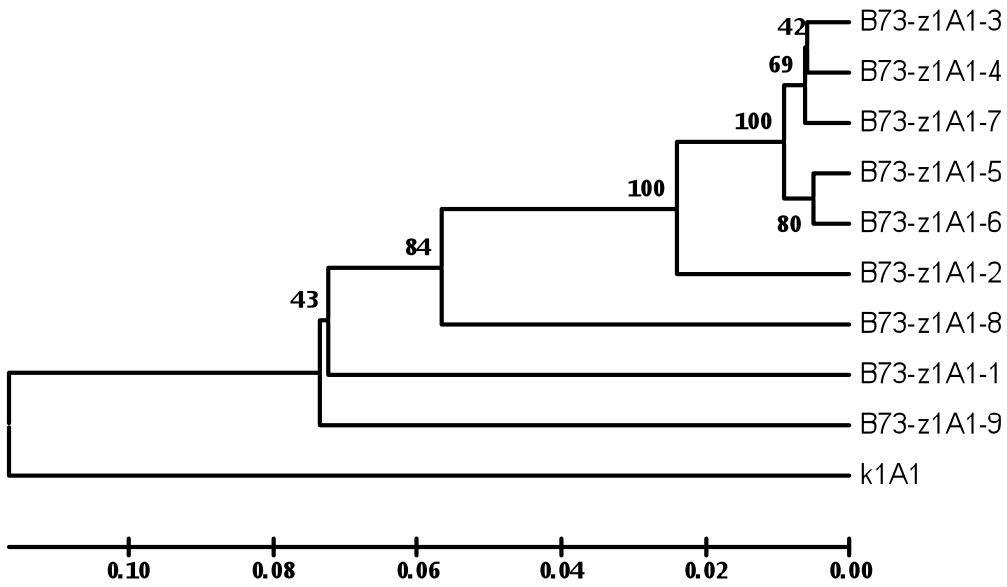

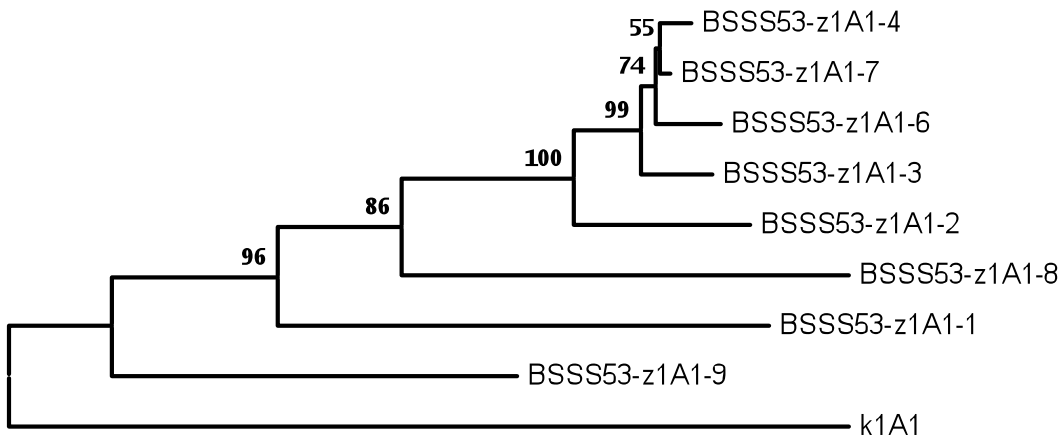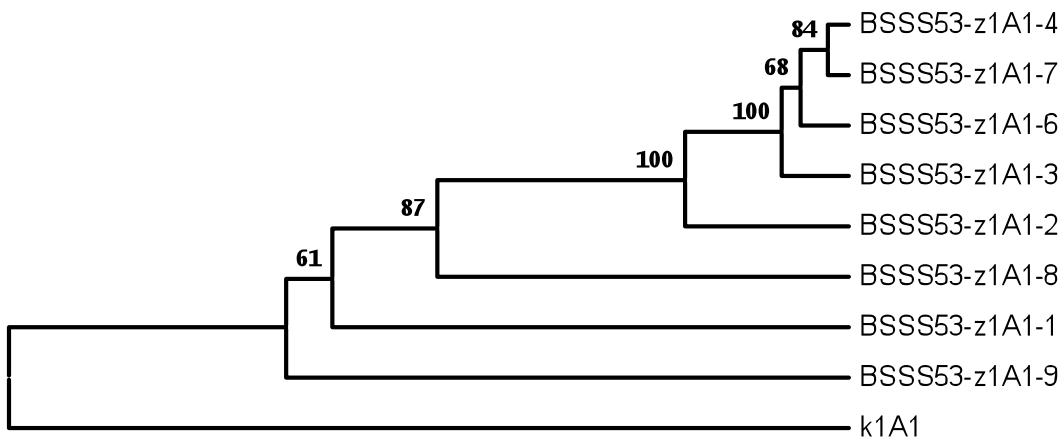

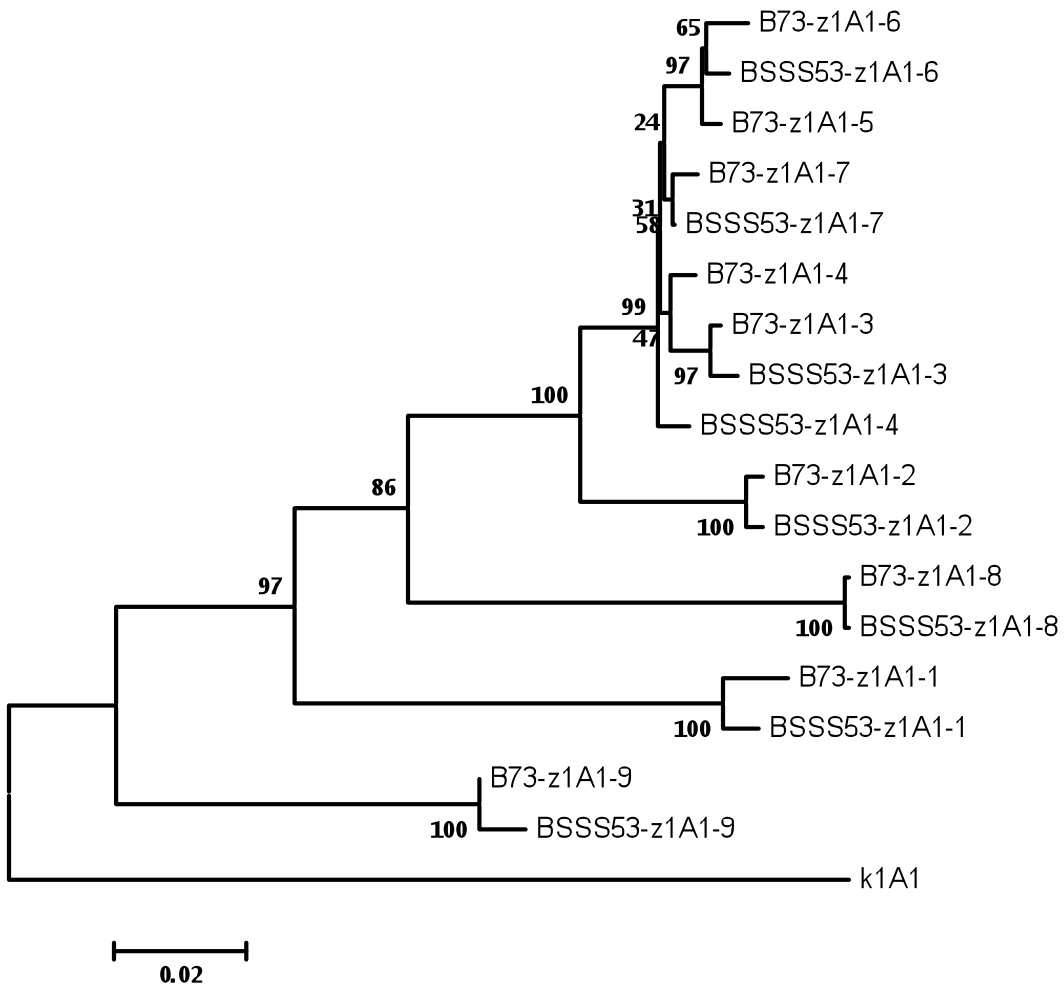

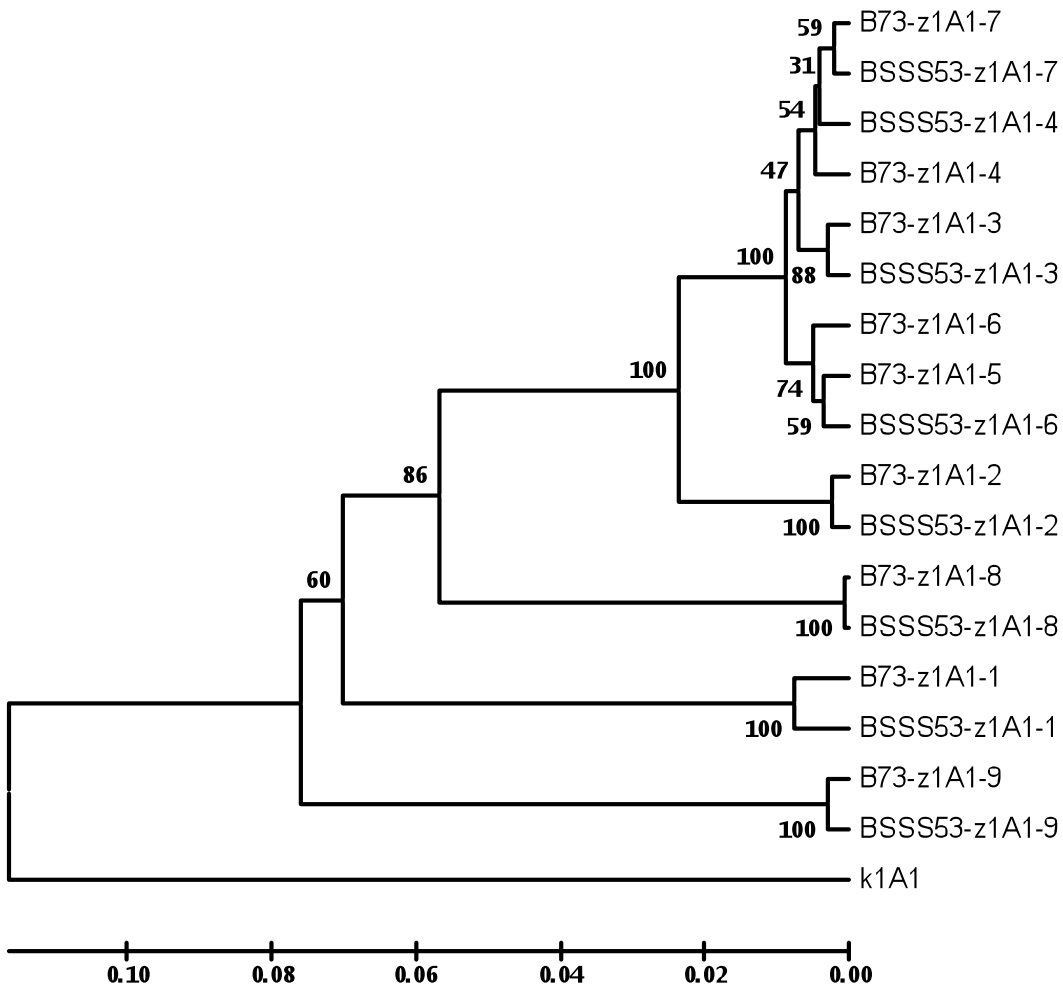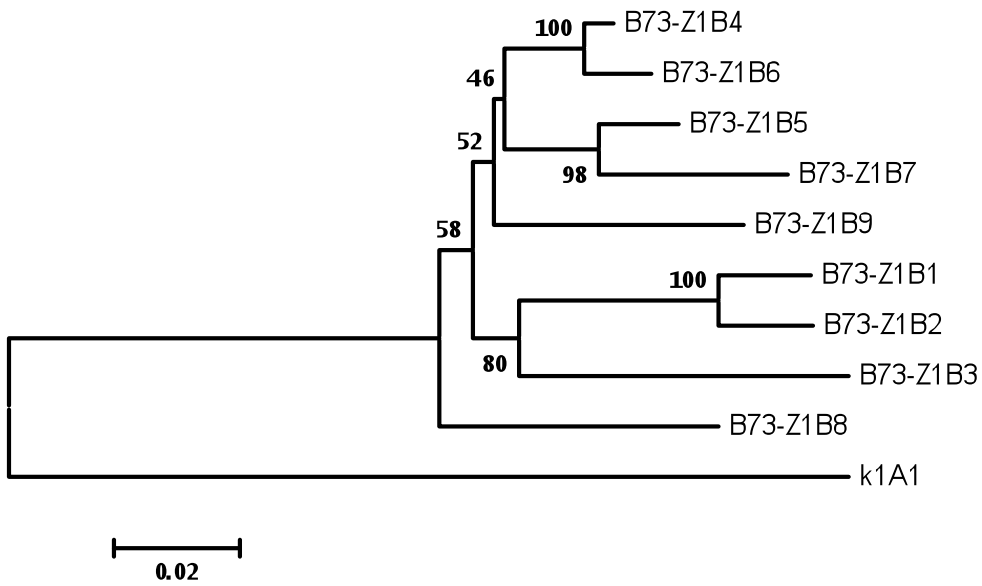

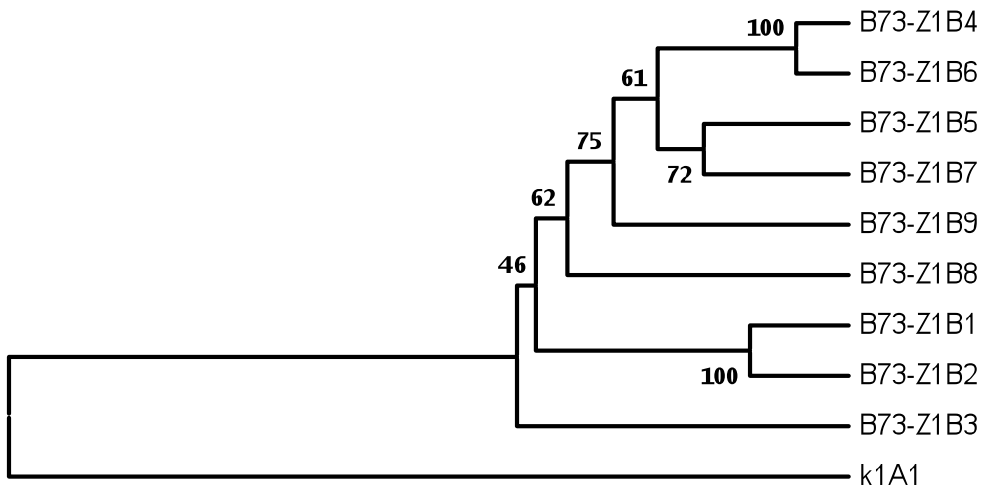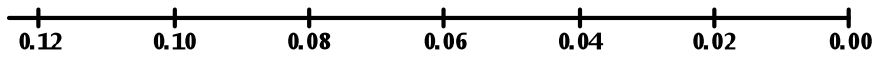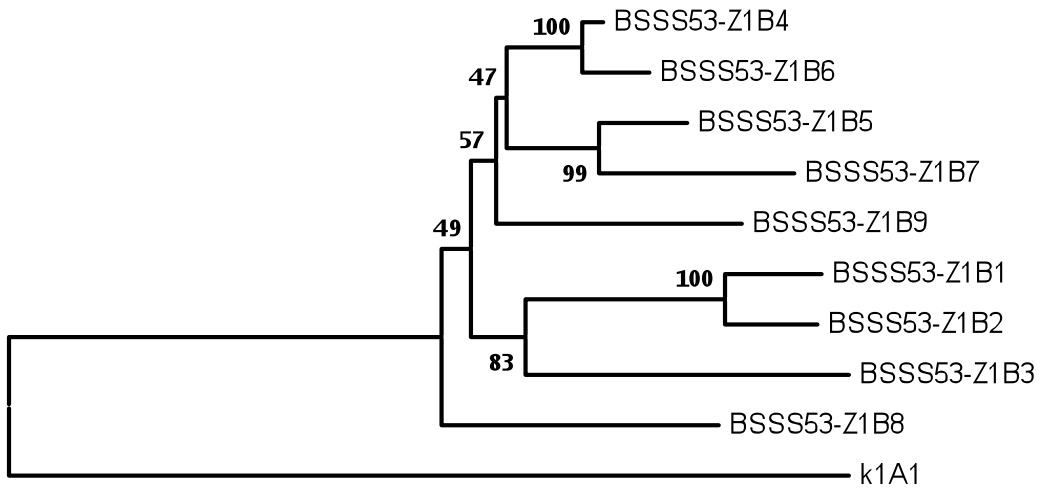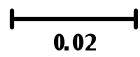

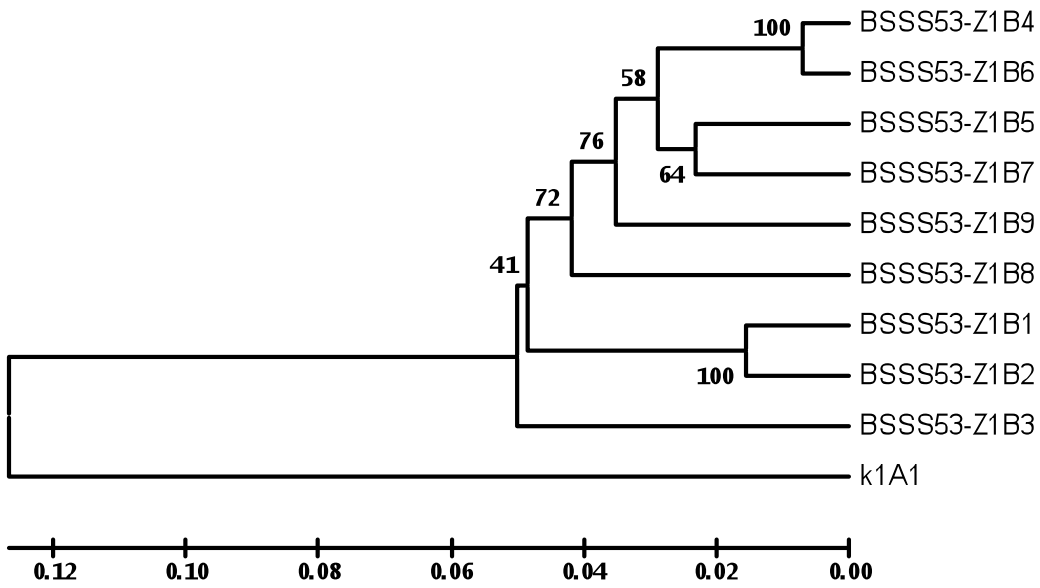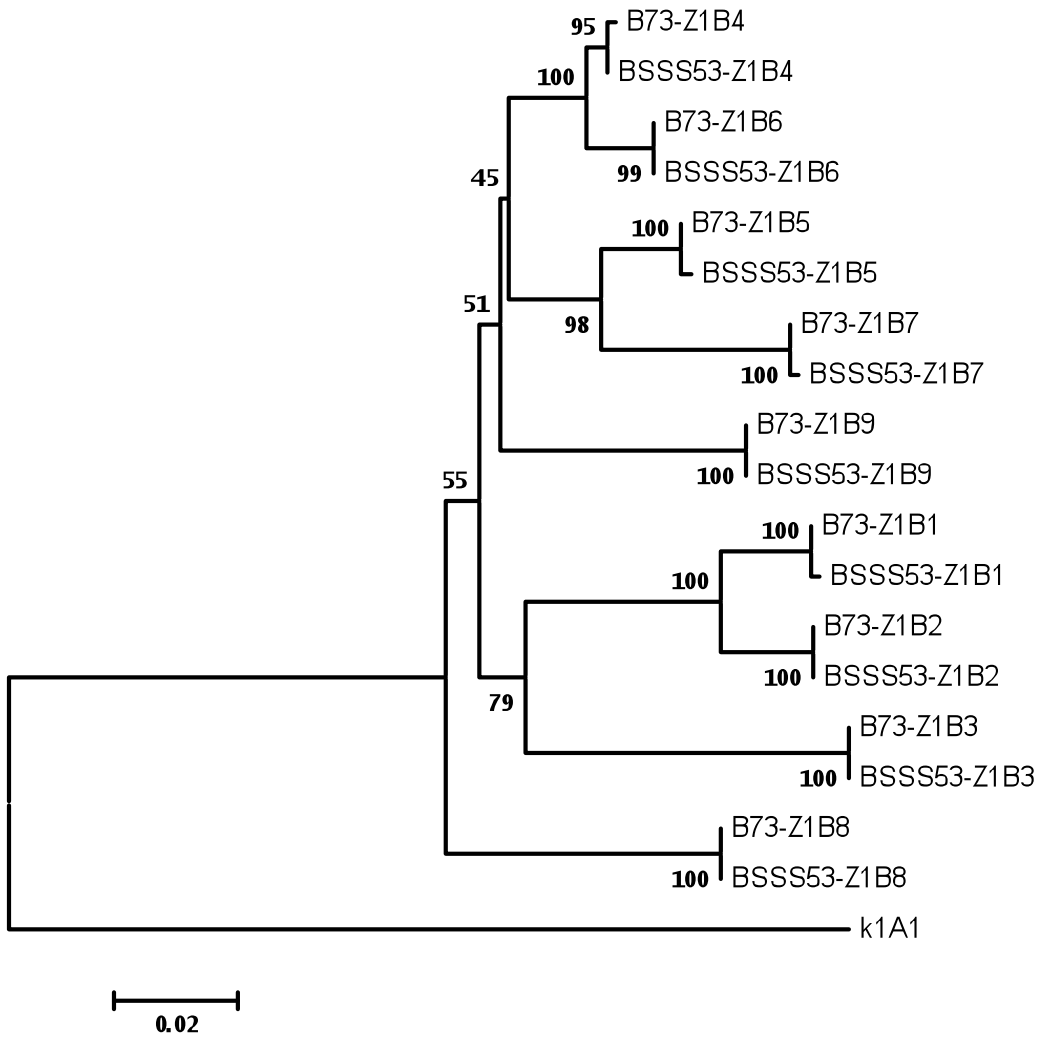

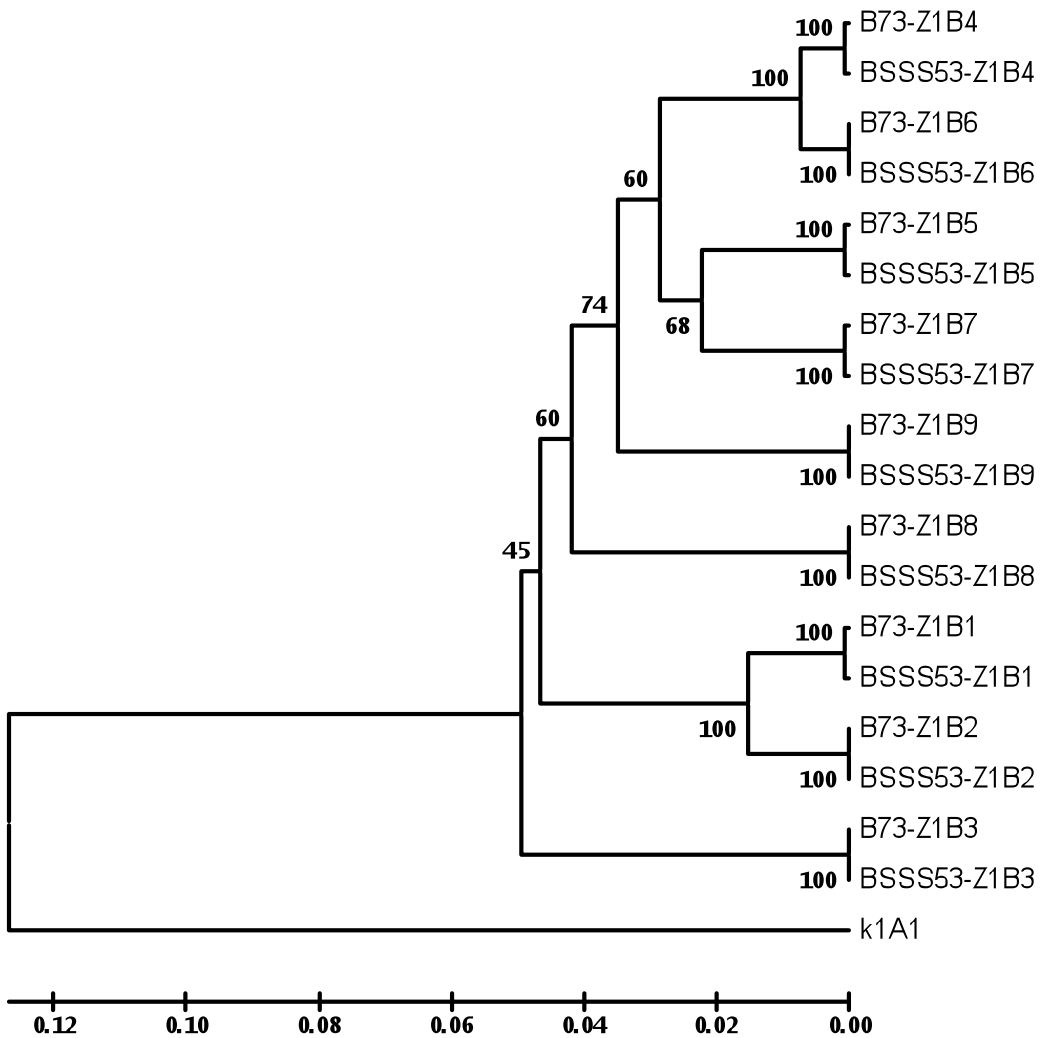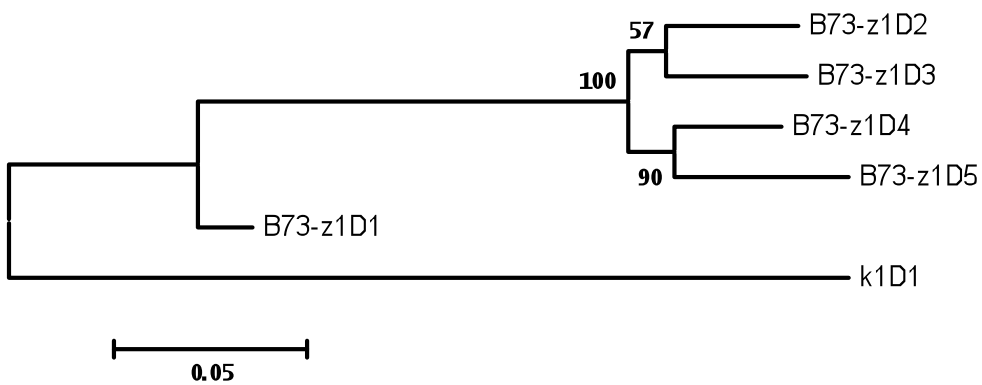

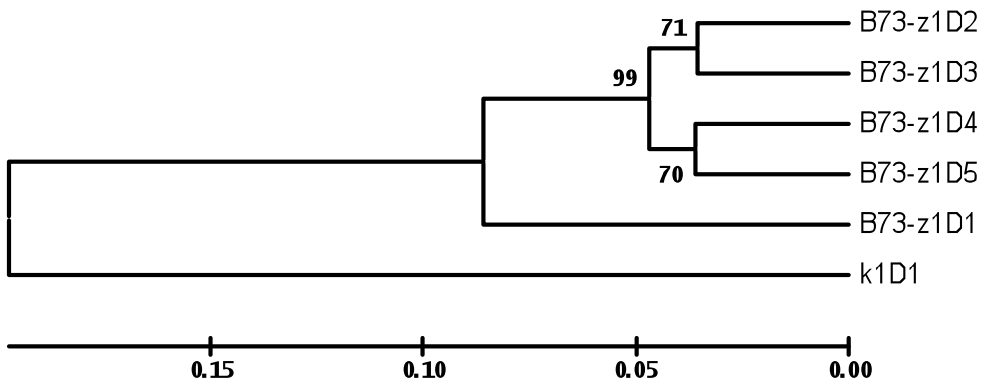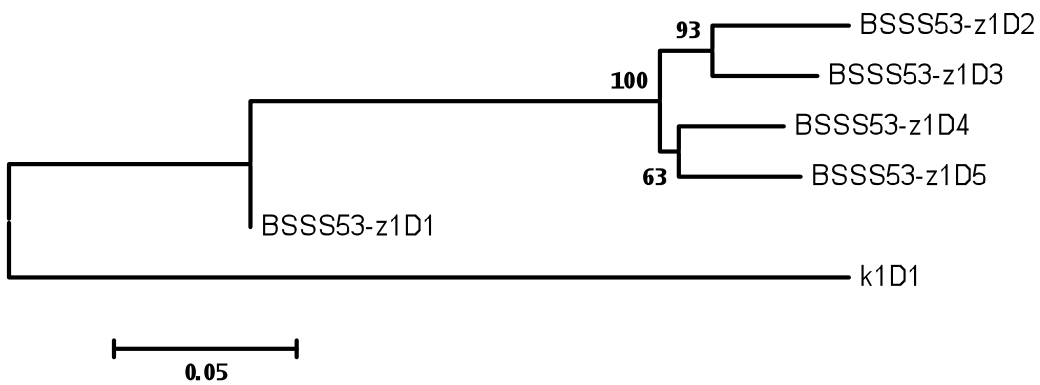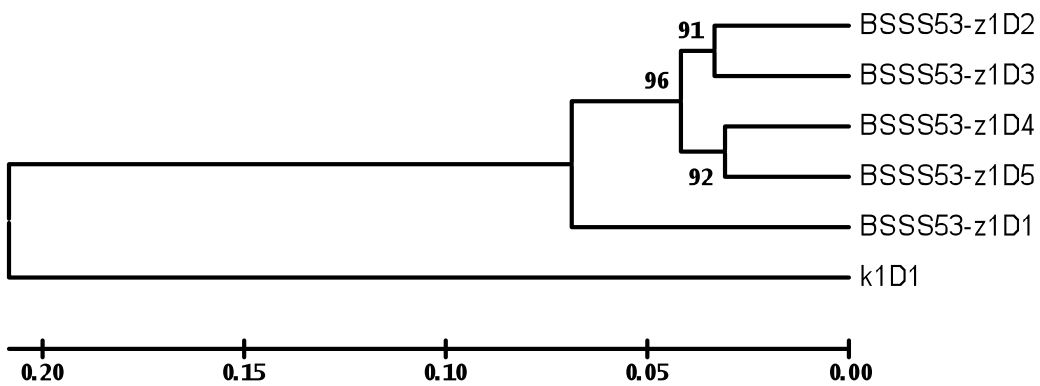

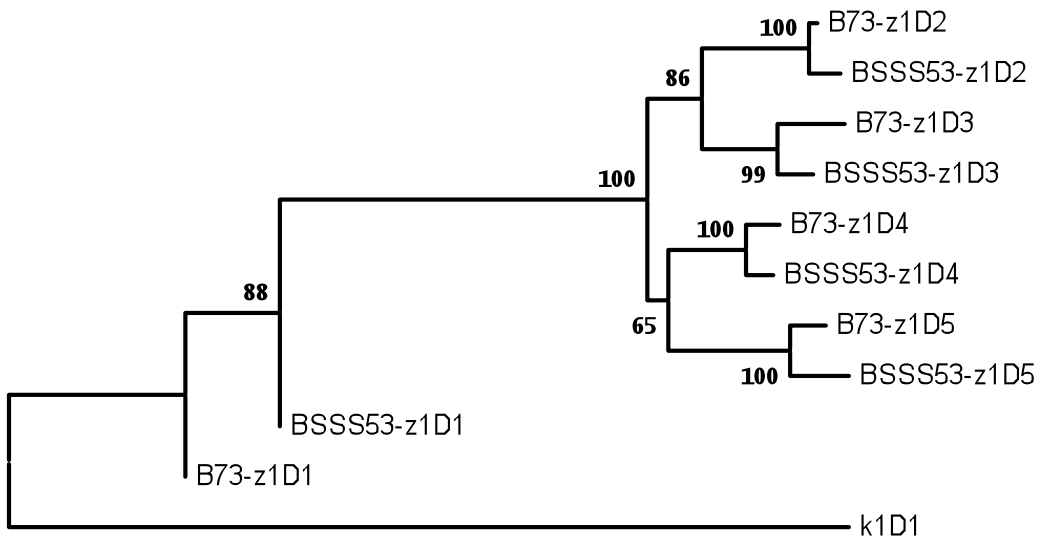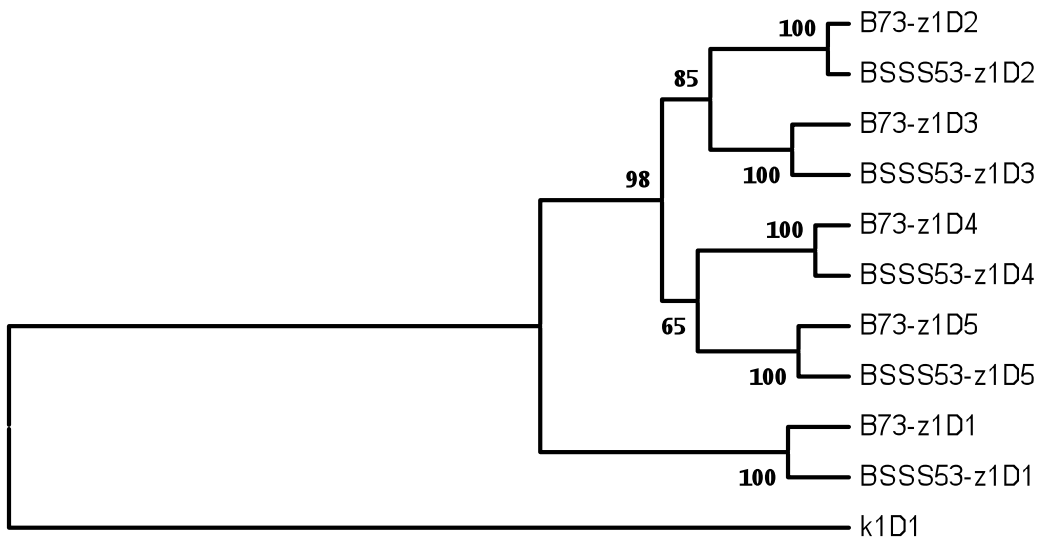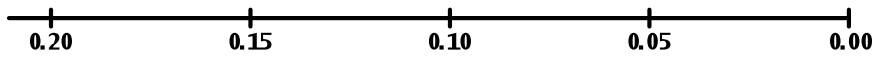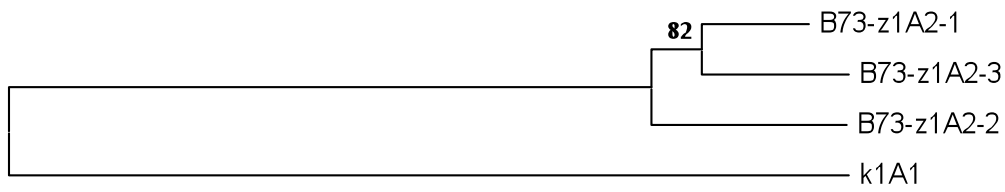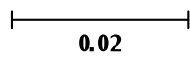

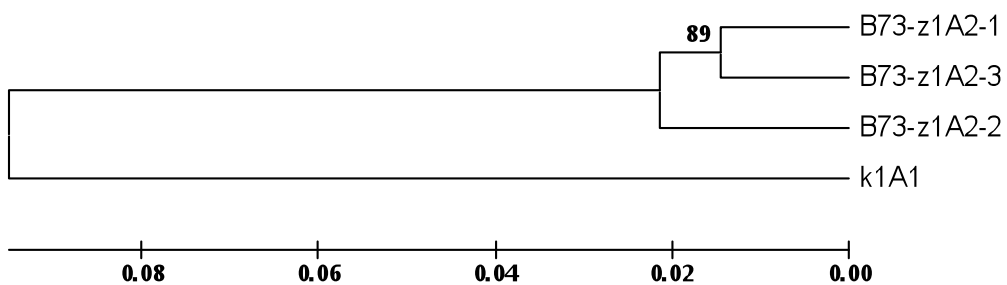

Supplement: Figure S1 — NJ and UPGMA trees constructed for each of the 19-kDa zein genes loci. At each locus an NJ tree was constructed for both inbred lines, followed by a UPGMA tree and then the same two methods were implied to construct a common tree with zein copies of both inbreds. We chose sequences of the prolamine genes in sorghum (kafirins) as outgroup. Genomic sequences were aligned using ClustalW and then Mega4 software was used to generate the trees. Bootstrap values are indicated on the branches of the tree, for 1,000 replicas. (PDF) [file pgen.1002131.s001.pdf]

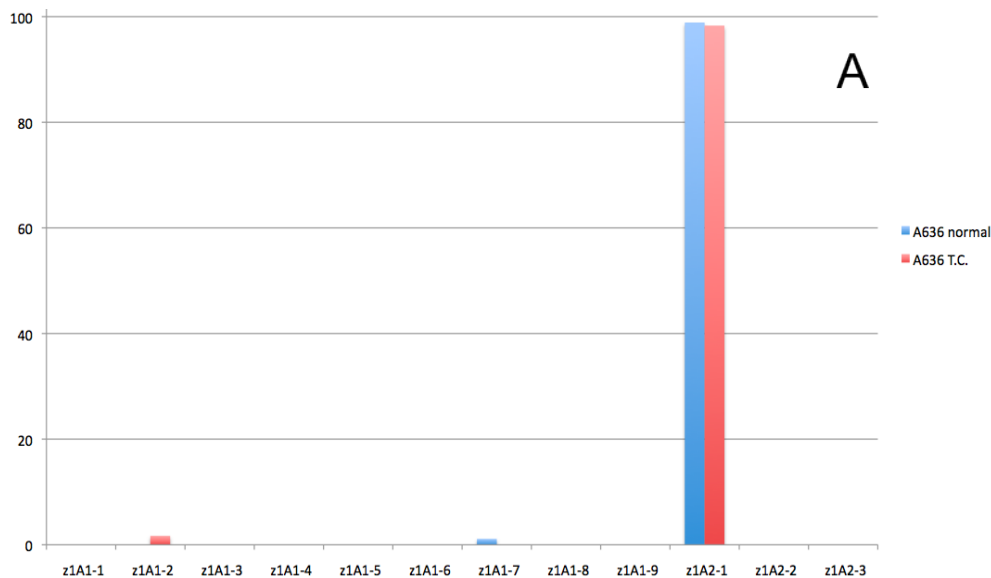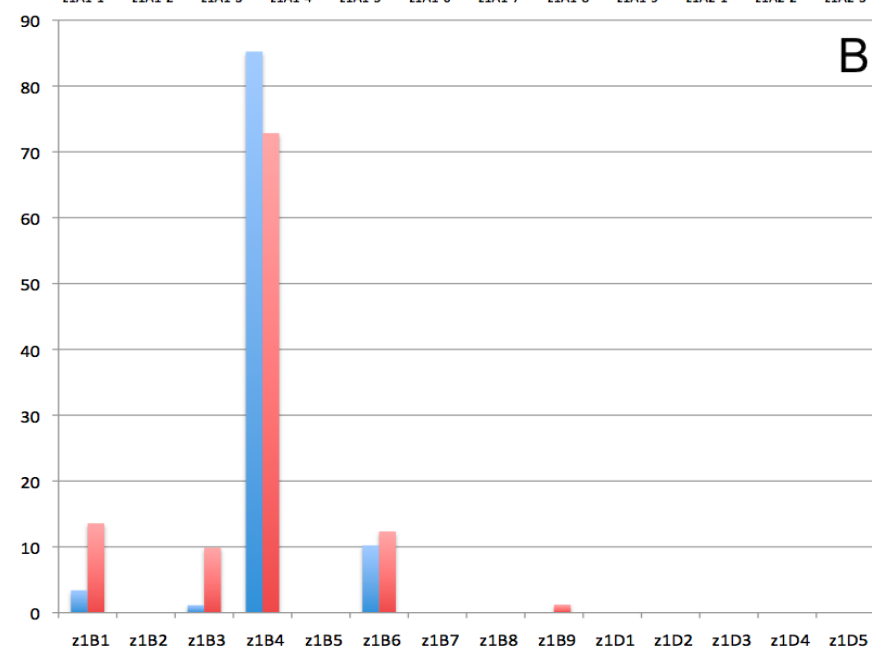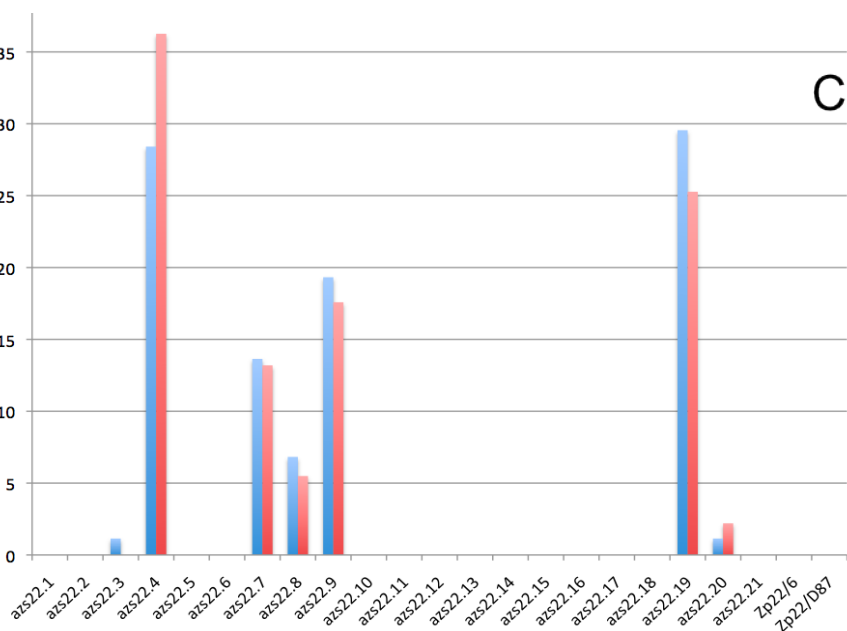

Supplement: Figure S3 — Extensive liquid tissue culture tends to re-establish the original methylation state. This is mainly visible for expression levels of copies at the z1C locus (C). Copies at the z1AI (A) and z1B/D (B) loci maintain the same pattern as in the original stages of tissue culture. (PDF) [file pgen.1002131.s003.pdf]

A

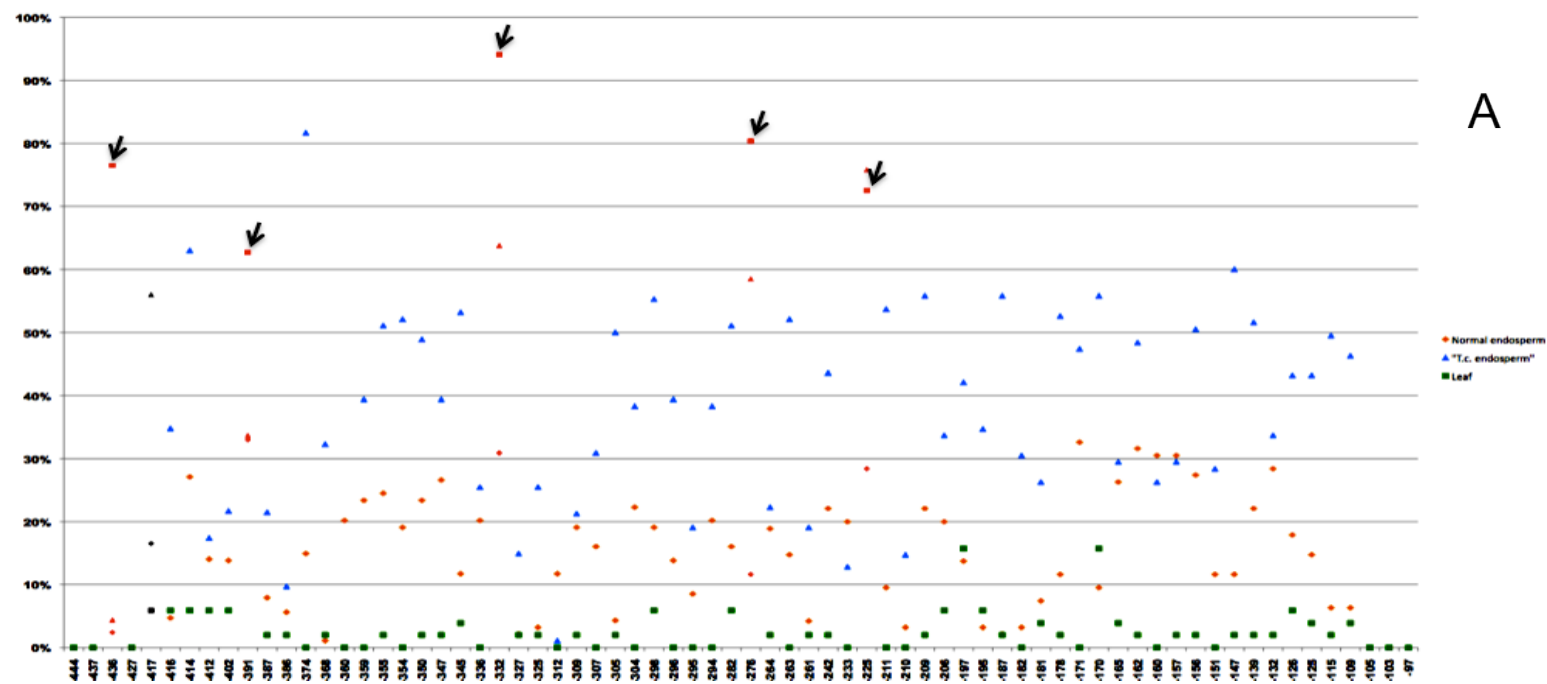

B

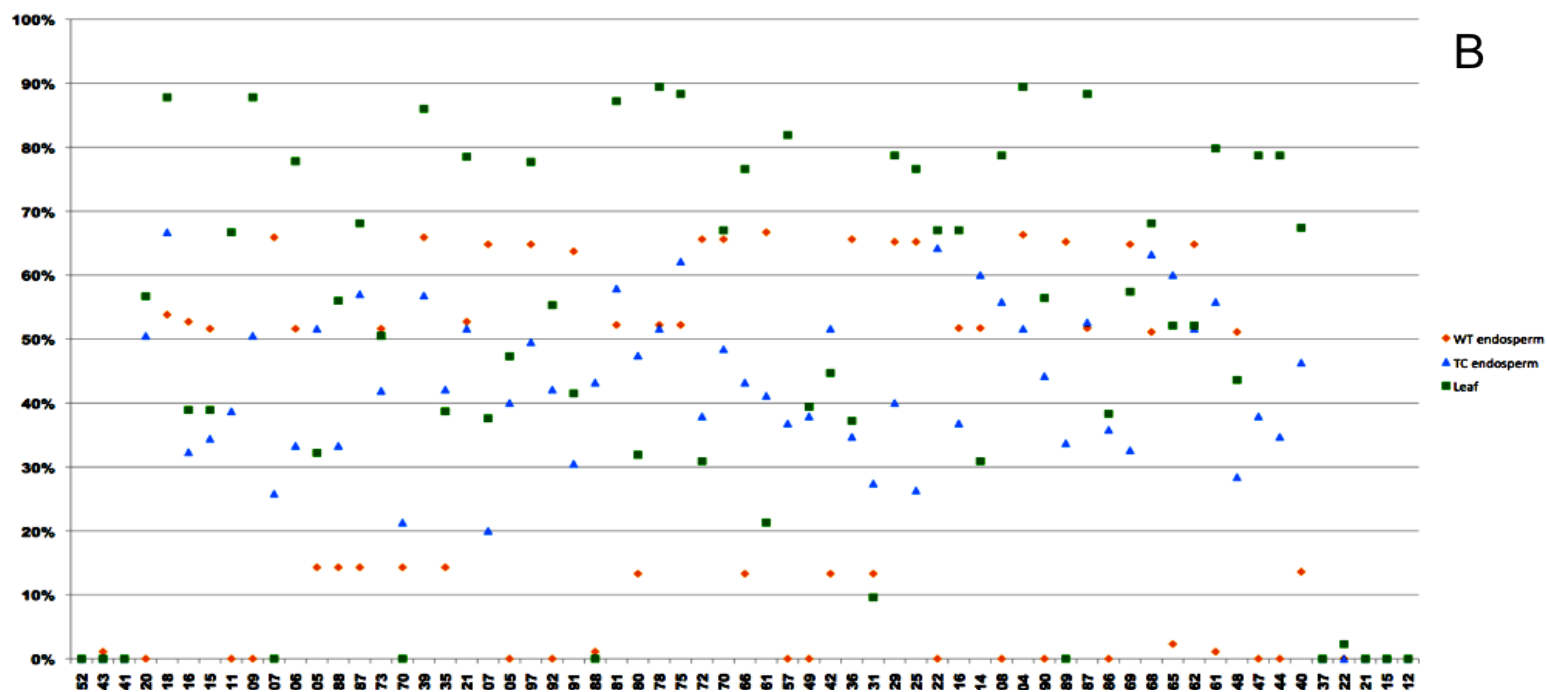

C

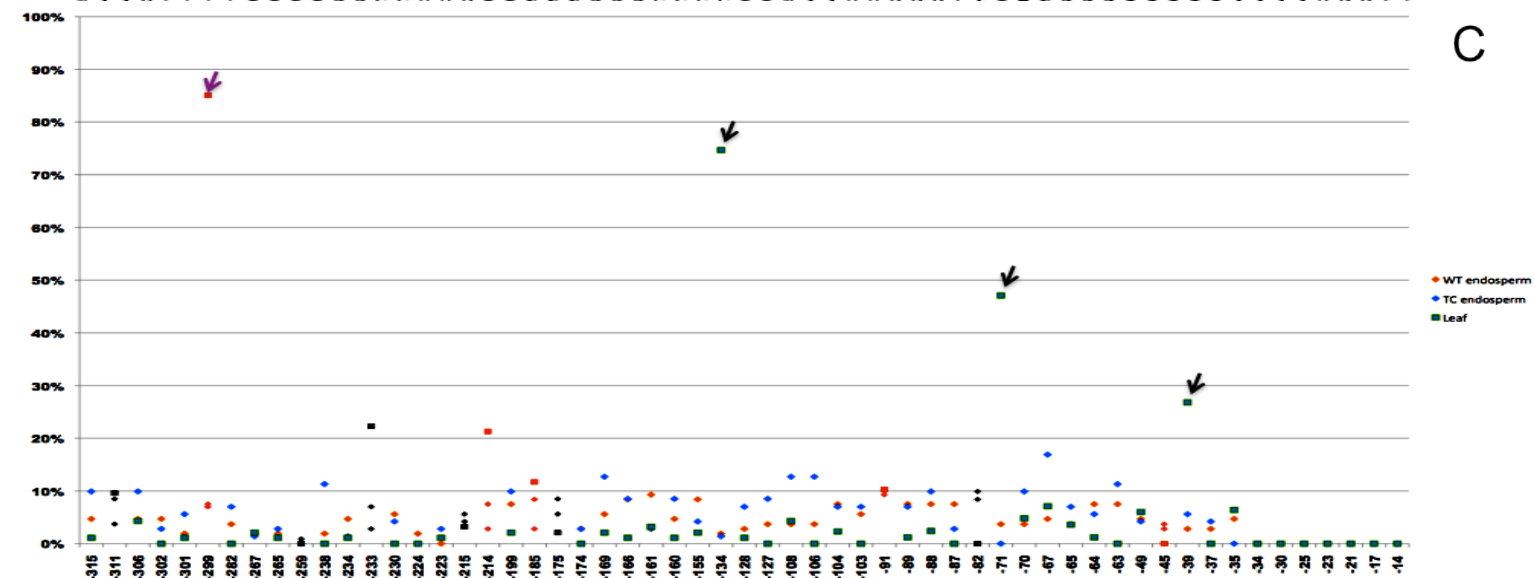

Supplement: Figure S4 — Methylation pattern in the promoters of z1A (A), z1B (B) and z1C (C). The positions of the cytosines present in the consensus sequence of each locus are marked relative to the start codon. Red label – CG methylation; Black label – CHG methylation; all the other data points are CHH methylation. (PDF) [file pgen.1002131.s004.pdf]
