# Supplementary material for: Differential Gene Expression and Epiregulation of Alpha Zein Gene Copies in Maize Haplotypes
Source: PLoS Genet. 2011 Jun 23;7(6):e1002131. doi: 10.1371/journal.pgen.1002131 (PMC3121756; doi:10.1371/journal.pgen.1002131)
Supplement: Figure S2 — P-box conservation in the promoter region of the 19 kDa α-zeins in both inbreds. Red letters indicate SNPs in the P-box sequence of several zein genes. (PDF) [file pgen.1002131.s002.pdf]

Zein copy	B73	BSSS53
z1A1-1	GTGTAAAG	GTGTAAAG
z1A1-2	GTGTAAAG	GTGTAAAG
z1A1-3	GTGTAAAG	GTGTAAAG
z1A1-4	GTGTAAAG	GTGTAAAG
z1A1-5	GTGTAAAG	GTGTAAAG
z1A1-6	GTGTAAAG	n/a
z1A1-7	GTGTAAAG	GTGTAAAG
z1A1-8	GTGTAAAG	GTGTAAAG
z1A1-9	GTGTAAAG	GTGTAAAG
z1A2-1	GTGTAAAG	GTGTAAAG
z1A2-2	GTGTAAAG	GTGTAAAG
z1A2-3	TTGTAAAG	TTGTAAAG
z1B1	GTGTGAAG	GTGTGAAG
z1B2	GTGTGAAG	GTGTGAAG
z1B3	GTGTGAAG	GTGTGAAG
z1B4	GTGTAAAG	GTGTAAAG
z1B5	GTGTAAAG	GTGTAAAG
z1B6	GTGTAAAG	GTGTAAAG
z1B7	GTGTAAAG	GTGTAAAG
z1B8	GTGTAAAG	GTGTAAAG
z1B9	GTGAAAAG	GTGAAAAG
z1D1	CTGTCTAA	CTAATCCAA
z1D2	GTGTAAAG	ATGTAAAG
z1D3	GTGTAAAG	GTGTAAAG
z1D4	GTGTAAAG	GTGTAAAG
z1D5	GCGTGAAC	GTGTAAAG
